# Heuristic Space Reduction Method for Source Localization in Water Distribution Networks

**DOI:** 10.1021/acsestwater.4c00671

**Published:** 2025-02-25

**Authors:** Gerardo Riano-Briceno, Ahmed Abokifa, Ahmad Taha, Lina Sela

**Affiliations:** † Fariborz Maseeh Department of Civil, Architectural and Environmental Engineering, The University of Texas at Austin, Austin, Texas 78712, United States; ‡ Department of Civil, Materials, and Environmental Engineering, University of Illinois Chicago, Chicago, Illinois 60607, United States; ¶ Department of Civil and Environmental Engineering and Department of Electrical and Computer Engineering, Vanderbilt University, Nashville, Tennessee 37235, United States

**Keywords:** source localization, water distribution systems, sensor fusion, heuristic optimization

## Abstract

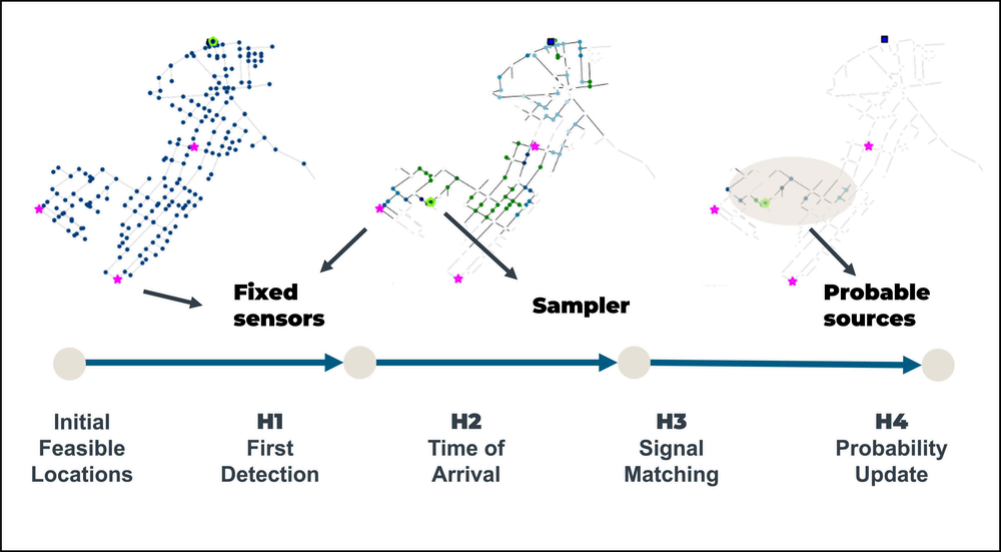

Ensuring water security and enabling timely responses to contamination events in water distribution systems (WDSs) rely heavily on the accurate and timely localization of contamination sources. Despite advances in water quality monitoring technologies, such as continuous sensing and grab-sampling, the coverage of monitoring remains sparse in most WDSs, making it difficult to accurately pinpoint the source of contamination. This paper introduces a novel source localization methodology designed to overcome these challenges by integrating sparse continuous sensing with targeted manual grab-sampling. The proposed approach iteratively narrows down the set of probable contamination sources by applying heuristics that account for the timing and signals from sensor measurements. To further address the uncertainty inherent in source localization, the methodology generates a probabilistic distribution over potential source locations. This distribution highlights areas requiring closer attention and guides where subsequent samples should be collected, effectively reducing uncertainty in the localization process. The methodology’s performance is validated through extensive analysis, demonstrating that combining fixed sensors with adaptive sampling significantly improves precision, accuracy, and localization speed, particularly in sparse sensor networks. The proposed approach advances the use of water quality sensing technology for source localization, with further research needed to optimize its effectiveness in improving WDS security and maximizing public health protection.

## Abstract 2

This research integrates continuous sensor and grab-sample data, using a probabilistic approach to improve accuracy and speed in source localization.

## Introduction

Water distribution systems (WDSs) comprise an intricate network of pipes that transport drinking water from sources and storage tanks to consumers. WDSs are crucial for supporting public health and sanitation, improving life quality, and promoting urban development.^1^ WDSs are vulnerable to both intentional and accidental contamination,^2,3^ as highlighted by recent waterborne disease outbreaks.^4−6^ These incidents highlight the critical need for precise source localization (SL) to identify contamination sources and limit further spread. Effective SL algorithms are crucial for minimizing impact following the detection of a contamination event, which can be identified by continuous quality sensors or grab-sample analyses.^7,8^ Despite advancements in sensor technology, the widespread implementation of continuous water quality sensors distributed in WDSs remains constrained by technical and economic challenges. As a result, reliable model-based decision support tools are essential to help utilities evaluate the potential benefits and justify investments in sensor systems. This paper proposes a model-based approach for SL, which explores the benefits of integrating continuous water quality sensors with grab-sampling.^9^

A significant body of research has addressed SL using simulation-optimization approaches. For example, SL has been formulated as an inverse problem, transforming transport differential equations into algebraic forms to construct an optimization program that minimizes the error between simulated and real data.^10^ Other studies have solved the optimization problem associated with SL using genetic algorithms.^11,12^ Also, the SL problem has been solved using an ensemble Kalman filter that relies on continuous water quality measurements to determine the true source despite uncertainty in sensor measurements.^13^ However, these methods often result in a single estimated source location, which can be inefficient if the actual source differs from the estimate. Moreover, the effectiveness of the simulation-optimization methods is significantly constrained by the accuracy of initial model assumptions, such as hydraulic parameters and demand patterns, where inaccuracies can lead to substantial deviations from actual contaminant distributions.^10,14^

Probabilistic SL approaches have demonstrated promising results in reducing errors caused by model uncertainty and expediting localization compared to simulation-optimization approaches.^15^ In probabilistic methods, source locations are assigned probabilities based on sensor data, with higher probabilities indicating a greater likelihood of being the source. Several authors proposed Bayesian frameworks incorporating backtracking^16^ for efficiency and iteratively updating the probability of feasible sources with sensor warnings, which characterize nodes as either clean or contaminated.^17,18^ Other researchers used Bayesian inference to localize the source with stochastic demands demonstrating that the actual source is often marked as highly probable.^19,20^

Despite the benefits of handling uncertainty offered by probabilistic methods, the effectiveness of all previously proposed approaches is significantly limited by their reliance on static quality sensors installed at fixed locations within WDSs, which may not provide comprehensive network coverage. This limitation is primarily due to sparse sensor placement driven by resource constraints, leading to limited observability and contributing to the ill-posed nature of the problem. An ill-posed problem is characterized by insufficient or inconsistent data, making it challenging to accurately localize contamination sources.^15,16^ As a result, the application of these methods is restricted, as both the location and number of sensors are critical to their success.^21^

Alternatively, several SL methodologies are based on grab-sample analyses to extend coverage. SL based on grab-sampling takes quality measurements at various locations and time intervals to infer the source location. For instance, Mann et al. proposed a source inversion algorithm with a simplified quality model that utilizes manual grab samples.^22^ Several works have also explored the impact of grab-sampling, using sensors that flow freely across the network and autonomous robots that take samples remotely, offering enhanced detection rates between 60 and 90% for short-duration injections in large-scale WDSs.^23−26^ Despite their wide coverage, grab-sampling methods are prone to human error, providing limited spatiotemporal data that may not reflect overall system conditions accurately.^27^ Additionally, these methods depend on increasing the number of samples to reduce uncertainty, which can be logistically challenging.^28^ Grab-sampling methods extend coverage and significantly reduce localization times, though they face challenges related to accuracy limitations, managing the number of samples, and determining where to sample next.^29,30^

This paper introduces a novel source localization (SL) methodology designed to address the challenges posed by existing approaches. First, we address the limitations of static quality sensors by integrating them with dynamic grab-sampling data, which enhances both network coverage and localization accuracy. Second, our approach mitigates the ill-posed nature of traditional SL problems by generating a probabilistic distribution over potential source locations, rather than identifying a single candidate source. The proposed method progressively refines the set of potential contamination sources by applying heuristics that incorporate the timing and characteristics of the measurements. Lastly, this probabilistic distribution identifies the key localization areas that require further attention and strategically guides where additional grab-samples should be collected, thereby effectively reducing uncertainty and improving the overall localization process.

The remainder of this article is organized as follows: First, we present the methodology and introduce the metrics used to evaluate localization performance. Next, we apply the method to a benchmark network under selected contamination scenarios. We then provide an exhaustive validation of the approach, exploring various configurations of continuous- and sampling-based sensing, along with a sensitivity analysis. Finally, we discuss the limitations of the proposed method and outline potential future directions for this research.

## Methods

The overall approach determines the source probability for each node in the WDS and updates it as new measurements are received, using four sequential heuristics to refine the likelihood of potential contamination sources. The process begins by eliminating locations that are not upstream of the initial contamination detection, based on flow paths. Then, sources are excluded if their time of arrival at the sensors does not align with the system’s flow patterns. The remaining nodes are then ranked according to their likelihood based on measurements. In the final stage, the probabilities for each node being the contamination source are updated based on the top-ranked nodes, and samplers are deployed to the locations with the highest probabilities to take additional measurements. This cycle repeats at the next sampling time, *t* + δ, incorporating new data to continuously improve the localization of the contamination source.

Next, we introduce the general definitions and notations used throughout this work. We then describe the offline and online stages of the method in detail. Finally, we define the metrics used to evaluate the method’s performance.

### Definitions

A WDS is represented by a graph , whose nodes  represent either demand nodes, reservoirs, tanks, or cross-connections between different hydraulic devices, and whose edges  represent pipes, pumps, and valves transporting water. The flow direction of an edge at time *t*, i.e., *q*_*e*_(*t*) ∈{−1, 1} is intrinsic to the edge’s notation. For example, describing an edge as *e* = (*n*_1_, *n*_2_) implies that flow moving from node *n*_1_ to node *n*_2_ is positive, i.e., *q*_*e*_(*t*) = 1. A negative flow direction is denoted with *q*_*e*_(*t*) = −1. Flow directions heavily influence the spread of contaminants in the network.

In this work, a contaminant is assumed to be injected in the WDS with baseline concentration *b*, at a node , from start time *t*_*o*_ to final time *t*_*f*_, thus lasting a specific duration *d* = *t*_*f*_ – *t*_*o*_. There are *N*_*c*_ optimally placed continuous quality sensors, referred to as *sensors* hereafter, taking continuous quality measurements at a subset of nodes , starting from a reference time *T*_*o*_ ≤ *t*_*o*_. Also, there are *N*_*m*_ operators, called *samplers* hereafter, performing manual grab-sampling. Samplers are deployed after the first detection ocurs, taking additional quality samples at specific times and nodes. The set of nodes at which samplers actively take and report quality measurements at time *t* is denoted as . We assume that both sensors and samplers monitor either the contaminant directly or other water quality parameters that are indicative of contamination. A sensor or sampler positioned at node *n* measures concentrations *c*_*n*_(*t*) ≥ 0 every δ hours. Detection occurs when a sensor or sampler first registers a contaminant concentration greater than the concentration tolerance ε_*c*_. The state of a node, *r*_*n*_(*t*) ∈ {0, 1}, is equal to zero if the node is clean, i.e., if *c*_*n*_(*t*) < ε_*c*_, and equal to one if it is contaminated, i.e., if *c*_*n*_(*t*) ≥ ε_*c*_. Concentration measurements at a node *n* from times *t*_1_ to *t*_2_ are described in compact vector form by ***c***_***n***_(*t*_1_: *t*_2_) = [*c*_*n*_(*t*_1_), *c*_*n*_(*t*_1_ + δ), ···, *c*_*n*_(*t*_2_))] ∈ _≥0_^(*t*_2_–*t*_1_)/δ^. The first detection is registered at time τ* when any of the installed sensors report a contaminated state at its location. The detection time for a sensor located at node *n* is expressed as τ_n_^*^.

### Offline Stage

To facilitate fast source localization, we create a reference database containing an exhaustive set of offline-simulated scenarios. Measurements from sensors and samplers are continuously compared to offline simulations, accounting for model and measurement uncertainties. Offline simulations start at a reference time *T*_*o*_ to minimize computational burden and have sensors in place before detection. We assume the contaminant injection can begin at any feasible start time , starting from reference time *T*_*o*_ to a final time *T*_*f*_ with steps of size δ, i.e., . This generates a set of feasible contamination scenarios, , based on injection baseline, duration, location, and start time.

Contamination event detection and sensor placement have been extensively studied, and for the offline stage, we adopt the modeling assumptions and sensor placement strategies from prior works.^31−33^ Contamination scenarios are simulated using EPANET^34^ through the WNTR Python package.^35^ For each feasible scenario , simulated contaminant concentrations at every node *n*, i.e., *c*_(*n*,*s*)_(*t*) are recorded for every time *t* ∈ [*T*_*o*_, *T*_*f*_] every δ hours, thus matching sensor sampling times. Likewise, detection times τ_(*n*,*s*)_^*^ are recorded for every sensor location *n* and every feasible scenario . Model uncertainty is introduced by randomizing demand patterns through sampling demands from a normal distribution. These simulations provide water quality values for potential contamination scenarios, which are later compared with real-time measurements to identify the most likely scenarios. A set of *N*_*c*_ sensors is optimally placed using the impact method^36^ implemented via the Chama Python package.^37^ This approach minimizes sensor detection times, considering budget constraints and contamination scenario characteristics.^37^ Once placed, sensors remain fixed at their assigned locations.

Reference start and end times must be carefully chosen and updated. The reference start time *T*_*o*_ corresponds to the time at which the analysis starts, i.e., when sensors start taking measurements. The final time *T*_*f*_ could be defined as the maximum water age in the network, which serves as a proxy of the maximum amount of time that a contaminant takes to spread from the reservoirs to the most downstream node in the network. Also, *T*_*f*_ could be chosen based on computational capacity, given that the longer offline simulations become, the higher their computational cost. Overall, start and final times are selected to represent a typical operating day of the water distribution system. If *T*_*f*_ is reached in real-time, it would be necessary to compute another round of offline simulations, with final time *T*_*f*_ becoming the new start time *T*_*o*_.

### Online Stage

**Figure 1:**
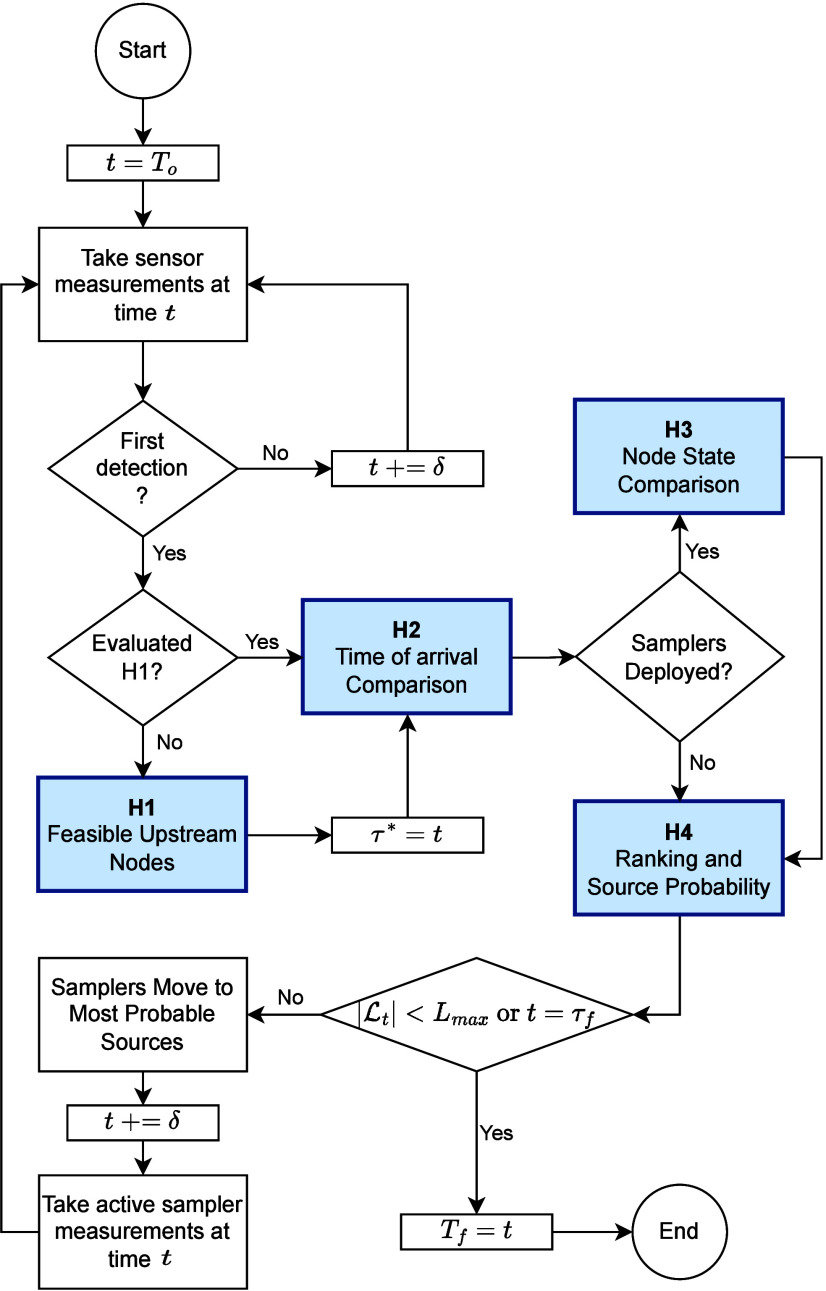
Heuristics to determine reduced feasible set of sources and probability update.

The online stage involves determining the source probability for every node in the system and reducing uncertainty by iteratively evaluating four heuristics. Heuristics are computed to determine which of the scenarios computed offline are feasible or not based on sensor measurements and grab-sample data. As heuristics are computed, scenarios are eliminated, thus reducing uncertainty about feasible source locations. Heuristics are labeled H1 through H4 and comprise the *computation of feasible upstream nodes* (H1), the *comparison of the time of arrival to sensors* (H2), the *comparison of sampler states* (H3), and the *rank and update probability* (H4). As shown in Figure 1, all heuristics are evaluated after the first detection occurs, i.e., at time *t* ≥ τ*. H1 is evaluated by discarding all scenarios whose source is not upstream of the sensor that first detected the contamination. Hence, H1 is only evaluated once. Then, H2 and H4 are evaluated iteratively, one after the other, every time a new sensor measurement or quality sample is gathered after the first detection. H3 is evaluated after H2, yet only after the deployment of samplers. The source probability is computed within H4. At the end of each heuristic evaluation, a new set of feasible scenarios  is determined for the next sampling time *t* + δ.

Samplers are deployed to locations with the highest source probability after the first evaluation of the H4 heuristic. Once they reach the sampling location, every sampler *m* takes periodic concentration measurements every δ_*m*_ hours and remains at the sampling location for the sampling period *d*_*m*_ ≥ δ_*m*_. Samplers transmit concentration data every time they take a new measurement. Sampler measurements are not continuous in time, given that they spend time moving between sampling locations. Therefore, H3 is only evaluated for samplers that completed data transmission by the time of evaluation. The cycle of evaluating heuristics, taking sensor and sampler measurements, stops when the number of feasible sources is less than a threshold *L*_*max*_ at time τ_*max*_ or after reaching a predetermined convergence time τ_*f*_ as shown in Figure 1. Throughout this process, the heuristics collectively discard sources deemed improbable based on observations from sensors and samplers, considering sampled concentrations, times of arrival, and flow directions while considering inherent uncertainty.

#### H1 Heuristic - Feasible Upstream Nodes

H1 identifies all nodes that are not upstream of the first detecting sensor and marks them as unfeasible, as these will unlikely be the source of contamination. Determining nodes that are upstream of the first detecting sensor involves checking the flow directions from times *T*_*o*_ to τ* every δ hours. With every set of flow directions at time *t* ∈ [*T*_*o*_, τ*], the WDS is represented by a directed graph . Each graph is traversed backward using depth-first search,^38^ starting from the node n* where the first detection took place, such that nodes upstream n* are recorded as feasible sources. Then, all sources  for which the source is not upstream n* are considered infeasible.

#### H2 Heuristic - Time of Arrival Comparison

H2 involves comparing the sequence of actual arrival times, i.e., first detection times, with simulated offline scenarios for each feasible scenario after H1. This approach is inspired by the successful application of a similar method for transient event detection, which relies on the sequence of arrivals.^39^ Specifically, H2 compares the actual detection times τ_n_^*^ with the simulated detection times τ_(*n*,*s*)_^*^ at every sensor location  and for every scenario . We account for measurement and modeling uncertainty in arrival times by introducing a time tolerance ε_*t*_, which indicates the sensitivity to time differences. Scenarios with expected arrival times that do not match the observed times, within the margin for uncertainty ε_*t*_, are discarded. In other words, a potential contamination event remains feasible if the observed arrival time is within ± ε_*t*_ of the simulated time. After the first detection, at each time *t*, we observe the state, i.e., clean or contaminated, of all sensors and identify detections. Then, we check three conditions: (C1) if a sensor has not detected the event and it is not expected to detect contamination based on the simulated events; (C2) if the sensor has not detected the event yet but is expected to detect it at a later time within some uncertainty range; (C3) if a sensor has detected the event and the time of detection is within a specified uncertainty range from the simulated detection time. We consider a scenario feasible if all sensors meet any of the three conditions. If H2 is evaluated for the first time, the conditions are evaluated for every scenario that was marked as feasible by H1. Later on, as shown in Figure 1, the set of feasible scenarios  for which H2 is evaluated corresponds to the set of scenarios marked as feasible by H4 at time *t* – δ. As a result, the feasible set  decreases monotonically and is updated by H2, producing a smaller set .

#### H3 Heuristic - Sampler State Comparison

H3 involves comparing the real sampler state *r*_*n*_(*t*) (recorded online) with the expected state *r*_(*n*,*s*)_(*t*) (simulated offline) for each scenario  and every node *n* where a sampler is actively transmitting data. Similar to H2, scenarios whose expected states do not match the observed states, given a margin for uncertainty in time ε_*t*_, are discarded. Expected sampler states *r*_(*n*,*s*)_(*t*_*i*_) are extracted from simulated scenarios  for every sampled node at time *t*, i.e., , for times *t*_*i*_ within a time window that starts at time *t* – ε_*t*_ and ends at time *t* + ε_*t*_. For a scenario to remain feasible, at least one of the expected sampler states must coincide with the real state. H3 only checks samplers actively taking measurements, thus skipping samplers moving between sampling locations at time *t*.

The margin of uncertainty for time in H3 must be adjusted to account for possible discrepancies in expected sampler states. Differences between real and expected state values are mainly attributed to model errors produced by uncertainty in demands and injection baseline. However, if the margin for uncertainty is too big, H3 will not filter many scenarios. If the margin is too small, many scenarios could be eliminated from the feasible set, even dismissing the real scenario. If the margin is carefully calibrated, H3 eliminates unrealistic scenarios, keeping the actual scenario feasible. With the H3 heuristic, the set of feasible scenarios resulting from the H2 heuristic evaluation is refined, producing an updated set .

#### H4 Heuristic - Ranking and Probability Update

H4 refines the previous source probability by ranking feasible scenarios based on how closely their expected measurements match actual observations from both the sensors and samplers. When the online stage starts, it is assumed that all nodes in the network have equal source probability, i.e., . After detection, H4 recalculates the source probability of all nodes based on the frequency with which they appear at the top of the ranking. Concretely, the computation of H4 is comprised of three steps. First, for every feasible scenario , the Euclidean distances between the real and expected sensor and sampler measurements are computed and added from detection to current time, i.e., ∥***c***_**(*n***,***s*)**_(τ*: *t*) – ***c***_***n***_(τ*: *t*)∥, resulting in the total error Δ*c*(*s*, *t*) at time *t* for scenario *s*. Sampler data is collected intermittently, making it necessary to compute the distance between actual and expected sampler measurements only for the times when measurements are taken. Second, the set of highly likely scenarios  is formed by taking the top *N*_*s*_ scenarios with the smallest total errors. Third, the prior source probability is updated for every node  by the normalized frequency in the set of highly likely scenarios .

The normalized frequency *p̂*_*t*_(*n*) is computed by counting the number of scenarios in  associated with the source location . For this, we compute the indicator function *I*_*s*_(*n*), which is equal to one if location *n* is associated with the scenario  and zero otherwise. The count is normalized by the total number of top scenarios *N*_*s*_. Therefore, the source probability is updated as follows: *p*_*t*+1_ = *αp̂*_*t*+1_(*n*) + (1 – α) *p*_*t*_(*n*), where , *S*_Δ_ = {*s*_*i*_ = (*b*, *d*, *n*, *t*): Δ*c*(*s*_*i*–1_, *t*) < Δ*c*(*s*, *t*) < Δ*c*(*s*_*i*+1_, *t*), *i* ≤ *N*_*s*_}, and α ∈ [0, 1] is a weighting factor. The updated probability *p*_*t*+δ_(*n*), at every node , is the weighted sum of the prior probability, i.e., *p*_*t*_(*n*), and the normalized frequency of the node *p̂*_*t*_(*n*). Updating the source probability in H4 resembles the exponential moving average (EMA), a statistical technique that smooths time series data by applying a weighting factor that decreases exponentially over time.^40^ EMA is particularly useful for maintaining the memory of past values while incorporating new observations. The smoothing factor α (0 < α < 1) controls the rate at which older data points decay in significance, balancing the influence of new data with historical trends.^40^

EMA is beneficial in this context because it allows the model to adapt to new data while retaining historical information. The higher the α, the more weight is given to recent observations, making the model more responsive to new data. Conversely, a lower α makes the model less sensitive to new observations, providing a smoother, more stable update. This approach is also advantageous because it ensures that the probability remains well-defined over time and that its updates are not overly influenced by a single, possibly erroneous, data point. By blending prior knowledge with new observations, EMA helps reduce the impact of noise and false positives, leading to more robust and reliable probability estimates.

#### Deployment of Samplers

After computing the H4 heuristic, samplers are strategically deployed in the water distribution system. Initially, samplers move to the locations with the highest source probability as determined by the H4 heuristic. When multiple samplers are deployed, they are assigned to the top probable locations nearest to each of them, ensuring that no two samplers share the same sampling location. After the first detection, it takes *d̅* hours for the samplers to reach their initial sampling locations from their base station. Upon reaching their designated locations, each sampler *m* remains stationary for a fixed duration *d*_*m*_, collecting samples at regular time intervals δ_*m*_. With each new measurement, samplers transmit the collected data for analysis. Once the fixed sampling period concludes, samplers receive updated information about the most probable contamination locations. Based on this updated information, samplers move to the newly identified locations to repeat the sampling process. As they move from one sampling location to another, samplers take the estimated shortest path, which is predetermined by a precomputed origin-destination matrix.

### Performance Metrics

We propose five metrics that collectively evaluate the accuracy, precision, and speed of source localization. These have direct implications for the water utility in terms of the resources required, such as teams needed to be dispatched in order to accurately pinpoint the source of contamination. The proposed five metrics include the *localization time*, the *number of localization areas*, the *largest localization area*, the *total localization effort*, and the *localization gap distance*.

#### Localization Time (LT)

**Figure 2:**
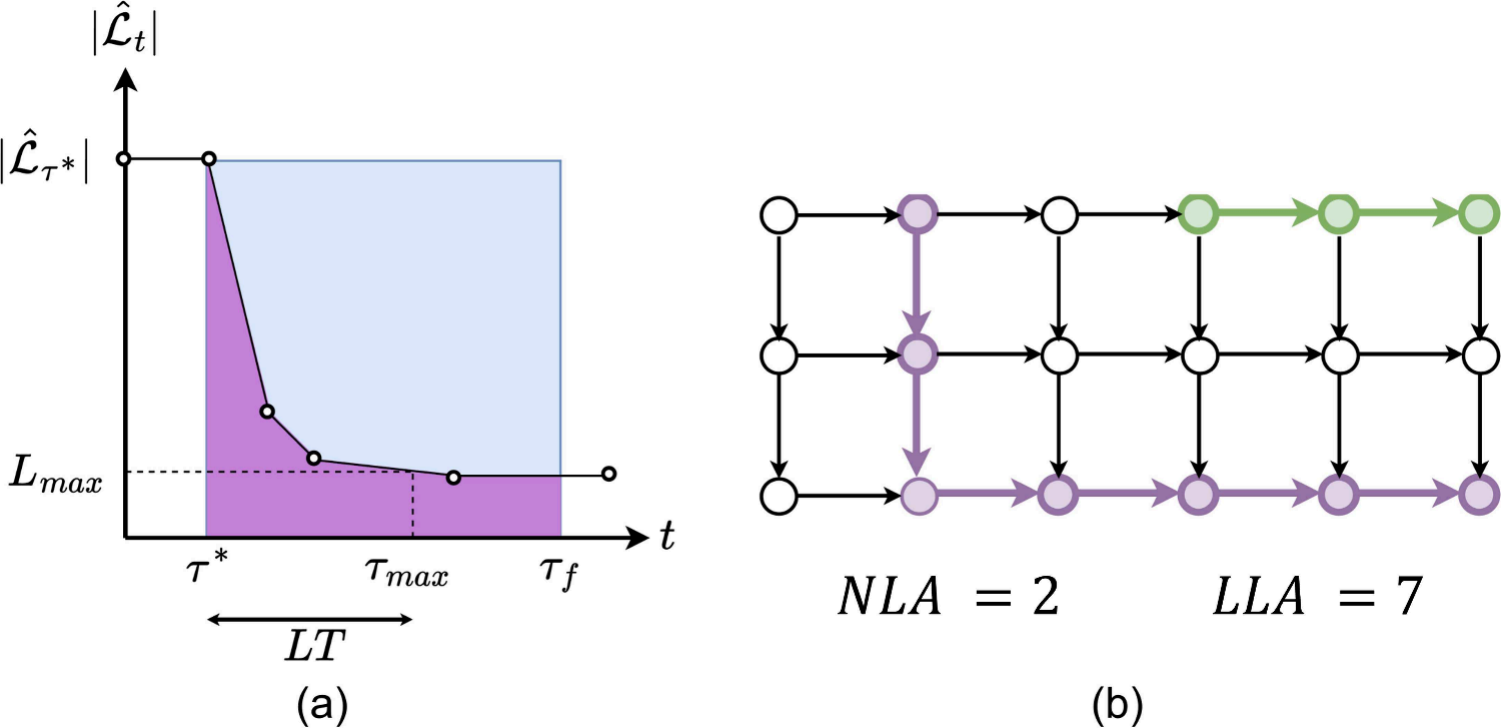
Illustration of the performance metrics: (a) Localization time (LT) and total localization effort (TLE) and (b) Number of localization areas (NLA) and largest localization area (LLA).

The LT index measures the time elapsed between the first detection, τ*, and the time, τ_*max*_, when the number of probable sources is less than a predefined threshold *L*_*max*_. We expect that as more samples are gathered, the variance in source probability *p*_*t*_ will decrease, meaning a small set of nodes will contain the majority of the source probability, with minimal probability assigned to the remaining nodes. This aligns with the Pareto Principle, which suggests that most effects come from a few causes.^41^ We define the set of probable sources  at time *t*, as the set of nodes that hold 90% of the total probability. For this reason, rather than setting a fixed convergence threshold based on probability, we use *L*_*max*_ to represent the maximum allowable number of probable sources. Water utilities can adjust *L*_*max*_ based on their performance targets, for example, specifying that the process should continue until fewer than 15 probable sources remain. If the threshold *L*_*max*_ is not reached during the SL method execution, we set LT to infinity. On the other hand, a higher threshold *L*_*max*_, results in shorter localization time. A smaller LT indicates better performance, as it reflects quicker source localization and a reduced extent of contamination. The LT index allows for comparisons across different scenarios and WDSs, provided the same threshold is used. The LT performance score is illustrated in Figure 2(a).

#### Number of Localization Areas (NLA)

The set of probable nodes may consist of one or several localization areas (i.e., connected components). A connected component in the WDS refers to the subset of probable nodes that are connected to each other by an edge in the graph  representing the WDS. Then, the set of connected components  is derived from  at time *t*. The NLA at time *t* measures the size of the set . To illustrate, in Figure 2(b), a WDS comprises 18 nodes interconnected by pipes forming a lattice structure. The probable nodes of the WDS are grouped in two localization areas, which are highlighted in green and purple. Therefore, for the WDS in Figure 2(b), the NLA is equal to 2. The NLA metric characterizes precision by quantifying how scattered the resulting set of probable sources is. The larger the NLA, the more scattered the resulting set of probable sources and the longer the time to localize the source, as many areas would need to be explored to pinpoint the source accurately.

#### Largest Localization Area (LLA)

The LLA index refers to the size of the largest localization area measured as the number of nodes in the localization area. For the example WDS in Figure 2(b), the largest localization area contains 7 nodes. Thus, the LLA is equal to 7. In general, larger LLA suggests that the contamination is potentially spread over a broader area, which implies longer localization times, as a greater number of probable locations must be explored to accurately identify the contamination source.

#### Total Localization Effort (TLE)

The TLE metric is the normalized area under the curve of the number of probable nodes that contain 90% of the source probability, i.e., , from the first detection time to a predetermined stopping time τ_*f*_ > τ* (see the area in magenta in Figure 2(a)), i.e.,  where δ is the sampling step size. Figure 2(a) shows the TLE in purple where the number of probable nodes decreases with time from the first detection τ* to the specified time horizon τ_*f*_. We normalize the TLE by the worst-case scenario area, which corresponds to the TLE as if the number of probable nodes would not be reduced (see the area in blue in Figure 2(a)). The normalization is done such that the TLE metric remains between 0 and 1, thus allowing the comparison between different scenarios and WDSs. Since the TLE describes an area over time, the TLE is not only proportional to the reduction in the size of  but also to the time it takes to reduce . Then, the TLE value describes how many feasible locations have been discarded by the method and reflects how fast the reduction is achieved. The smaller the TLE, the higher the method’s precision and the faster its convergence.

#### Localization Gap Distance (LGD)

If the true source is not within a localization area, the LGD measures the distance from the true source to the nearest probable source, calculated as the number of pipes along the shortest path. If the true source is within the set of probable sources  at time *t*, the LGD is zero; otherwise, it is greater than zero. A smaller LGD indicates a more accurate estimation, as the true source is closer to the set of probable nodes.

## Results and Discussion

**Figure 3:**
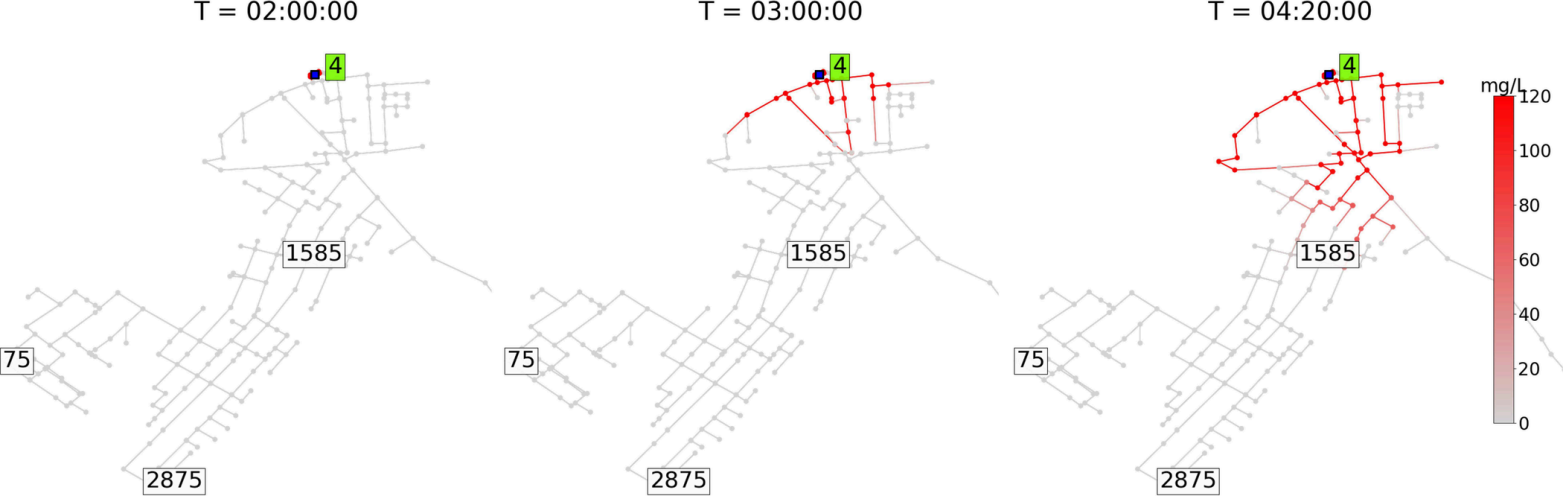
Propagation of contaminant in PA2 network for scenario A.

In this section, we (i) demonstrate the proposed approach using selected scenarios, (ii) evaluate its performance with exhaustive simulations, and (iii) assess the robustness of the proposed approach using sensitivity analysis, considering factors such as initial sensor placement and uncertainties in time of arrivals. We use the PA2 network depicted in Figure 3, consisting of 262 junctions, 288 pipes, one reservoir, and one pump, to demonstrate our approach. The PA 2 system is based on a portion of the Cheshire Distribution system near Harrisburg, PA.^42^ The system hydraulics and water quality are simulated with EPANET, modeling contaminant injections as first-order decay reactions.^34^ The simulations employ a hydraulic time step of 20 min and a quality time step of 5 min. Model error is introduced by randomizing demand patterns, achieved by sampling parameters from a normal distribution centered around the nominal parameter value, with a standard deviation equal to 20% of the mean. The methodology and assumptions related to baseline concentration and injection duration are based on the modeling settings from previous studies on contaminant event detection.^31−33^ Sensors are placed using the Chama Python package, developed by Sandia National Laboratories.^37^ For all scenarios, δ = δ_*m*_ = 20 min, *d*_*m*_ = *d̅* = 60 min, *T*_*o*_ = 0, *T*_*f*_ = 35 h, τ_*f*_ = 8 h, and ε_*t*_ = 1 h. The proposed algorithm and all simulations were implemented and executed using Python.

### Illustrative Analysis

We use four contamination scenarios to illustrate each step of the algorithm and analyze performance when events are detected by one, two, and three sensors. This highlights how factors such as localization challenges, detection time, along with the process of updating the size of the localization and source probabilities. We show detailed results for scenario A, and the results for scenarios B-D are shown in the Supporting Information (SI).

#### Nonuniqueness in Contamination Events

We illustrate four scenarios involving contaminant injections at nodes ’4’, ’139’, ’43’, and ’155’, with a baseline concentration of 120 mg/L and a 7-h duration. Three static sensors gathering continuous real-valued contaminant concentrations are optimally placed in the network at nodes ’1585’, ’75’, and ’2875’. For each scenario, detecting sensors, start times, and detection times are shown in Table 1. For scenario A, all sensors detect contamination, whereas two out of three sensors detect the contamination for scenario B, and one for scenarios C and D.

**Table 1:** Parameters for Selected Scenarios

**ID**	**Source**	**Detecting Sensors**	**Start Time**	**Detection Time**
A	’4’	’1585’, ’75’, ’2875’	02:00	04:20
B	’155’	’75’, ’2875’	04:00	10:20
C	’139’	’2875’	03:00	08:20
D	’43’	’1585’	01:00	08:40

**Figure 4:**
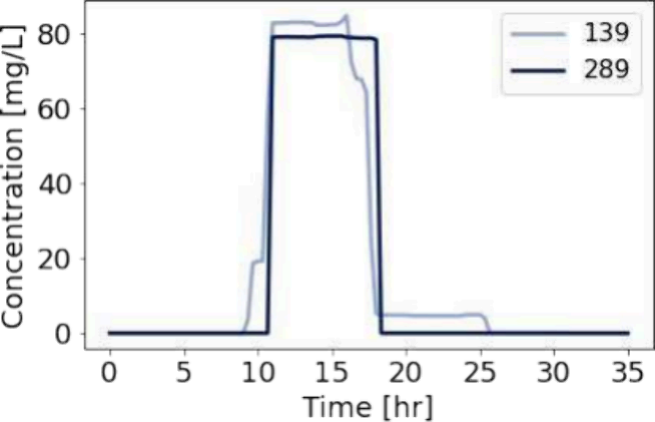
Measurements of contaminant concentration at node ’2875’ for 2 different contamination scenarios at nodes ’139’ and ’289’ starting at 3:00 and 10:00, respectively.

The frames in Figure 3 show how the contaminant propagates across the WDS in scenario A, starting from the source at node 4. Figure 3 includes quality results from the injection start time (most-left frame) to the first detection time (most-right frame). The color scale of the pipes in Figure 3 indicates the contaminant concentration level, ranging from no contamination, shown in gray, to higher concentrations marked in red. Variability in contamination spread across the different scenarios occurs due to the variability of flow velocities across the network. For example, although the first detection times are similar for scenarios C and D, contamination footprints are significantly different (see Figures S5 and S9 in the Supporting Information). On the other hand, the similarity in sensor measurements across different scenarios further complicates SL. For example, Figure 4 shows two similar signals observed at sensor ’2875’, despite the events originating from different locations and times. This variability in flow patterns, coupled with overlapping sensor responses from different source locations, underscores the complexity and ambiguity of the SL process, emphasizing its inherently ill-posed nature.

#### Localization Areas and Probability Update

**Figure 5:**
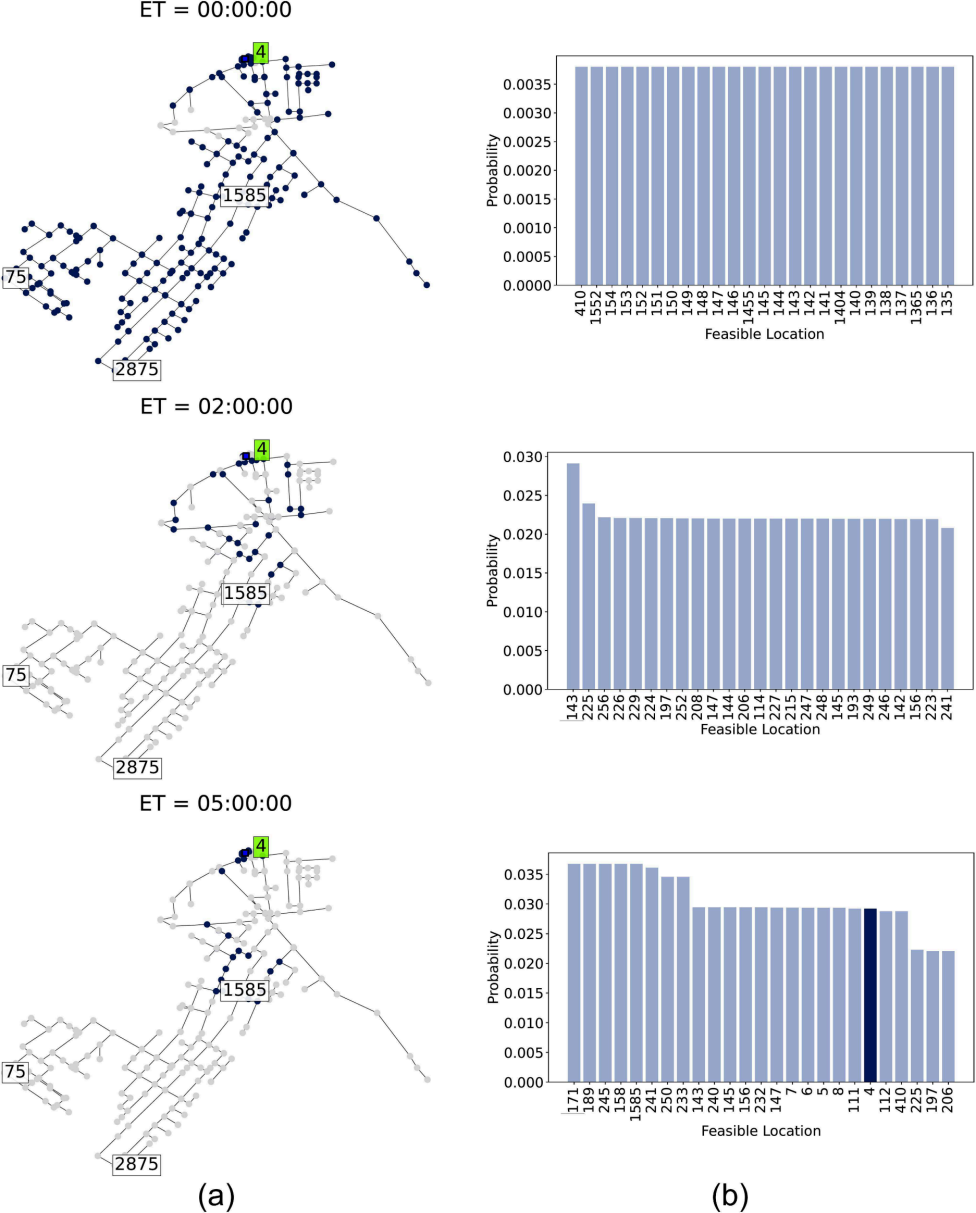
Evolution of the localization process with sensors in scenario A: (a) localization set with feasible sources marked in dark blue, and (b) source probability, with the true source highlighted in dark blue. ET refers to the elapsed time from first detection.

**Figure 6:**
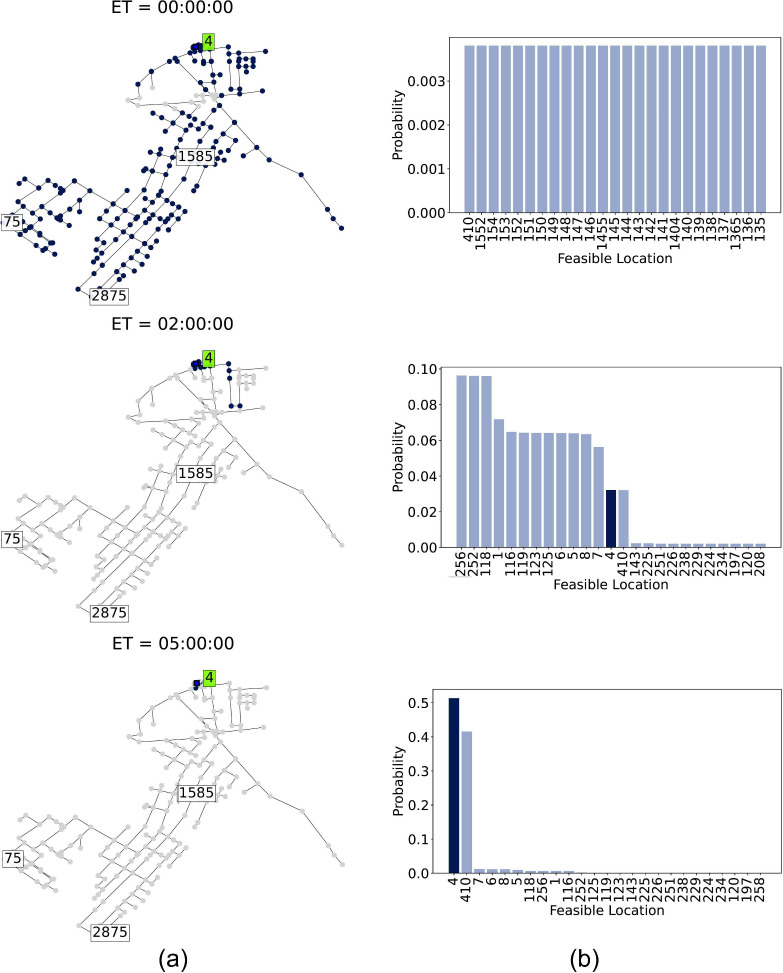
Evolution of the localization process with sensors and a sampler in scenario A: (a) localization set with feasible sources marked in dark blue, and (b) source probability, with the true source highlighted in dark blue. ET refers to the elapsed time from first detection.

Next, we demonstrate the evolution of the localization set and source probability in scenario A, comparing scenarios with and without a sampler. The remaining scenarios are shown in Figures S1–S12 in the Supporting Information. Results for the case without samplers is shown in Figure 5, and with samplers in Figure 6. These figures illustrate the evolution of the localization set and source probability from initial detection, showing observations two and five hours after the first detection (elapsed time (ET) is indicated in each frame). In Figures 5 and 6, the left frames (a) show the feasible source locations over time, and the right frames (b) display the probability distribution for the most likely source locations. For clarity, only the top 25 nodes with the highest probability are shown. Frames are ordered from the earliest at the top to the latest at the bottom. The true source, node ’4’, is highlighted in dark blue.

Implementing the SL algorithm with a sampler in scenario A leads to a quicker convergence toward a more precise set of probable sources near the contamination origin. Sources near sensor ’1585’ are rapidly marked as infeasible due to discrepancies between sampler measurements and simulations associated with these locations, as depicted in Figure 6(a). After initial detection, source probabilities are updated, and the sampler moves to the highest probability location. The sampler conducts measurements every 20 min and remains onsite for one hour, before relocating to the next most probably site. Despite intermittent and delayed samples, the use of a sampler allows the true source to quickly rise to the top of the probability rankings within two hours of detection. By the five-hour mark, variance is significantly reduced, narrowing the probable sources to just two key nodes: ’4’ (the true source) and ’410’ (upstream of ’4’), as shown in Figure 6(b) bottom. Without a sampler, however, the true source only becomes prominent five hours after detection, with the probability distribution remaining broadly spread among many potential sources. This results in a less precise set of probable sources, forming clusters around the true source and near sensor ’1585’ (Figure 5(a) and Figure 5(b) bottom). Although three sensors detect the contamination, sensors ’75’ and ’2875’ provide delayed actionable data. Thus, the sampler enhances precision and accelerates convergence by making additional measurements available shortly after the initial detection.

#### Performance Evaluation

**Figure 7:**
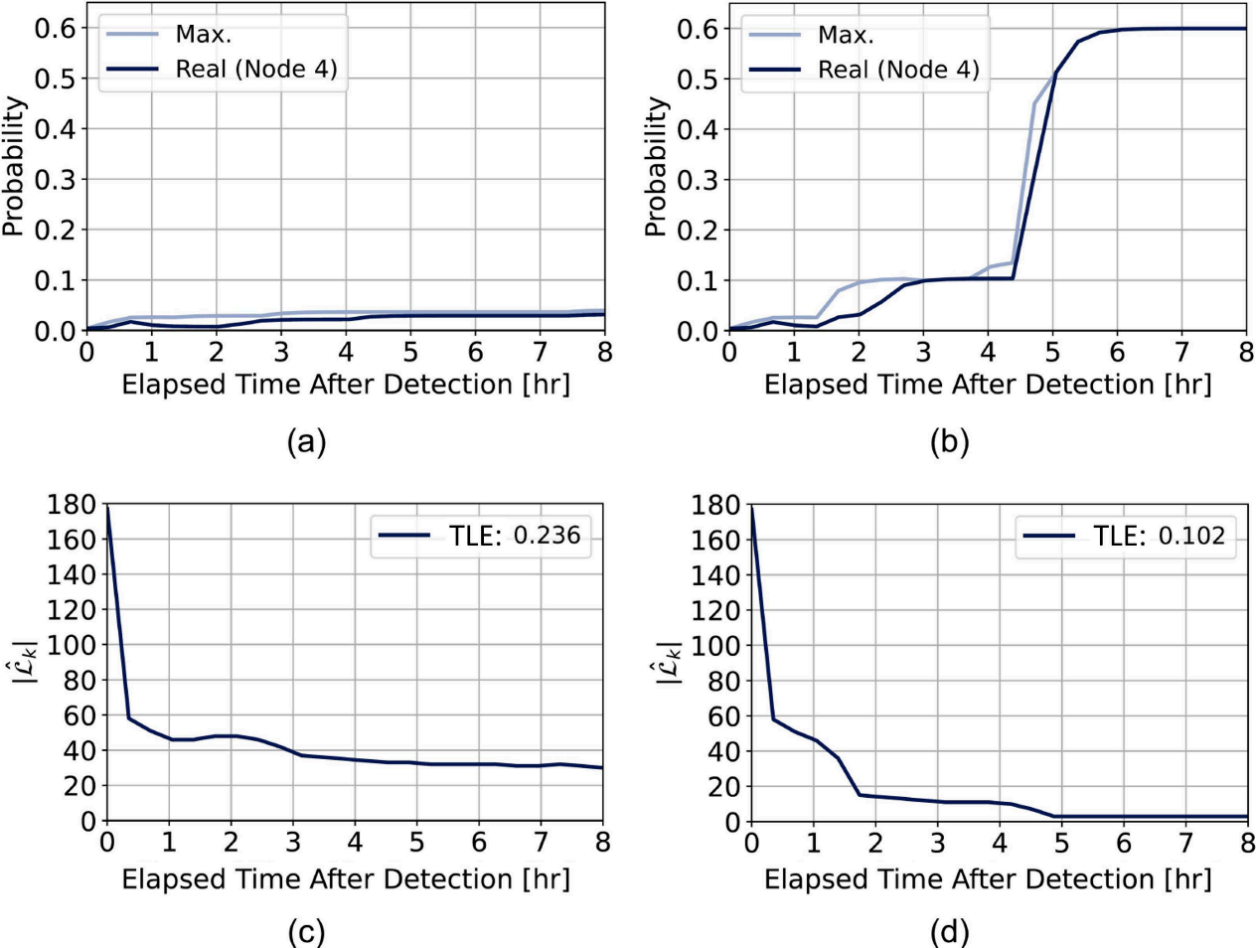
Comparison of true source probability vs maximum (a) with and (b) without a sampler in scenario A; (c) and (d) show the most probable nodes without and without a sampler.

We showcase the evolution of the source probability, the localization effort, and evaluate the five performance metrics. Figure 7 shows the source probability for both the true source and the maximum source probability over time, alongside the number of probable nodes that contain 90% of the source probability, , from the first detection time τ* to τ_*f*_ = τ* + 8 *hr*. Figures 7(a) and 7(b) illustrate how the source probability compares to the maximum source probability over time, highlighting that as more data is gathered, the probability of the true source converges toward the maximum. Figure 7(a) presents the scenario without a sampler, and Figure 7(b) with a sampler, demonstrating the impact of the sampler on the probability trajectory. When incorporating sampler data, the variance of the source probability decreases significantly, concentrating the probability in fewer locations and, thus, pinpointing the true source with higher accuracy. The maximum probability in Figure 7(a) is notably smaller compared to Figure 7(b), indicating that using a sampler increases accuracy and precision in locating the true source.

Figures 7(c) and (d) illustrate the changes in  and the localization effort over time for scenario A, comparing cases without and with a sampler. As expected, the Total Localization Effort (TLE) is smaller with a sampler (0.102) than without one (0.236), indicating more efficient convergence. Although the scenario without a sampler reaches a plateau in probable nodes faster, it remains less precise and shows no further improvement. In contrast, using a sampler allows the algorithm to reduce the feasible set of locations from 175 to just 14 within two hours of the first detection, demonstrating greater precision and faster convergence.

Table 2 lists the five performance metrics, including TLE, NLA, LLA, LGD, and the localization times until the number of probable sources is less than 35 and 15 (*L*_*max*_ = 40 and *L*_*max*_ = 30). Scenarios with and without a sampler are denoted by ’-S’ and ’-NS’, respectively. Despite variations in sensor measurements, the SL algorithm consistently identified the true source within the feasible sets by the final time step, τ_*f*_, as indicated by a LGD of zero in all scenarios. The other metrics, however, varied significantly. For instance, scenario A experienced the longest localization times, the largest TLE and LLA values at τ_*f*_, reflecting a more complex and extensive search space. In scenario A, the inclusion of a sampler reduced localization times dramatically, with the method converging to 40 and 30 feasible nodes in 1.33 and 1.67 h, respectively, compared to 3 and 7.67 h without a sampler. While localization times remained unchanged in scenarios B, C, and D when using samplers, there was a notable improvement in TLE, NLA, and LLA metrics across these scenarios.

**Table 2:** Performance Metrics for Selected Scenarios

**ID**	**LT [hr]** (*L*_*max*_ = 40)	**LT [hr]** (*L*_*max*_ = 30)	**TLE**	**NLA**	**LLA**	**LGD**
A-NS	3.00	7.67	0.236	3	16	0
A-S	1.33	1.67	0.102	1	1	0
B-NS	0.33	0.33	0.106	1	2	0
B-S	0.33	0.33	0.089	1	2	0
C-NS	0.33	0.33	0.122	3	8	0
C-S	0.33	0.33	0.096	2	4	0
D-NS	0.33	0.33	0.071	2	5	0
D-S	0.33	0.33	0.062	1	4	0

### Exhaustive Analysis

**Figure 8:**
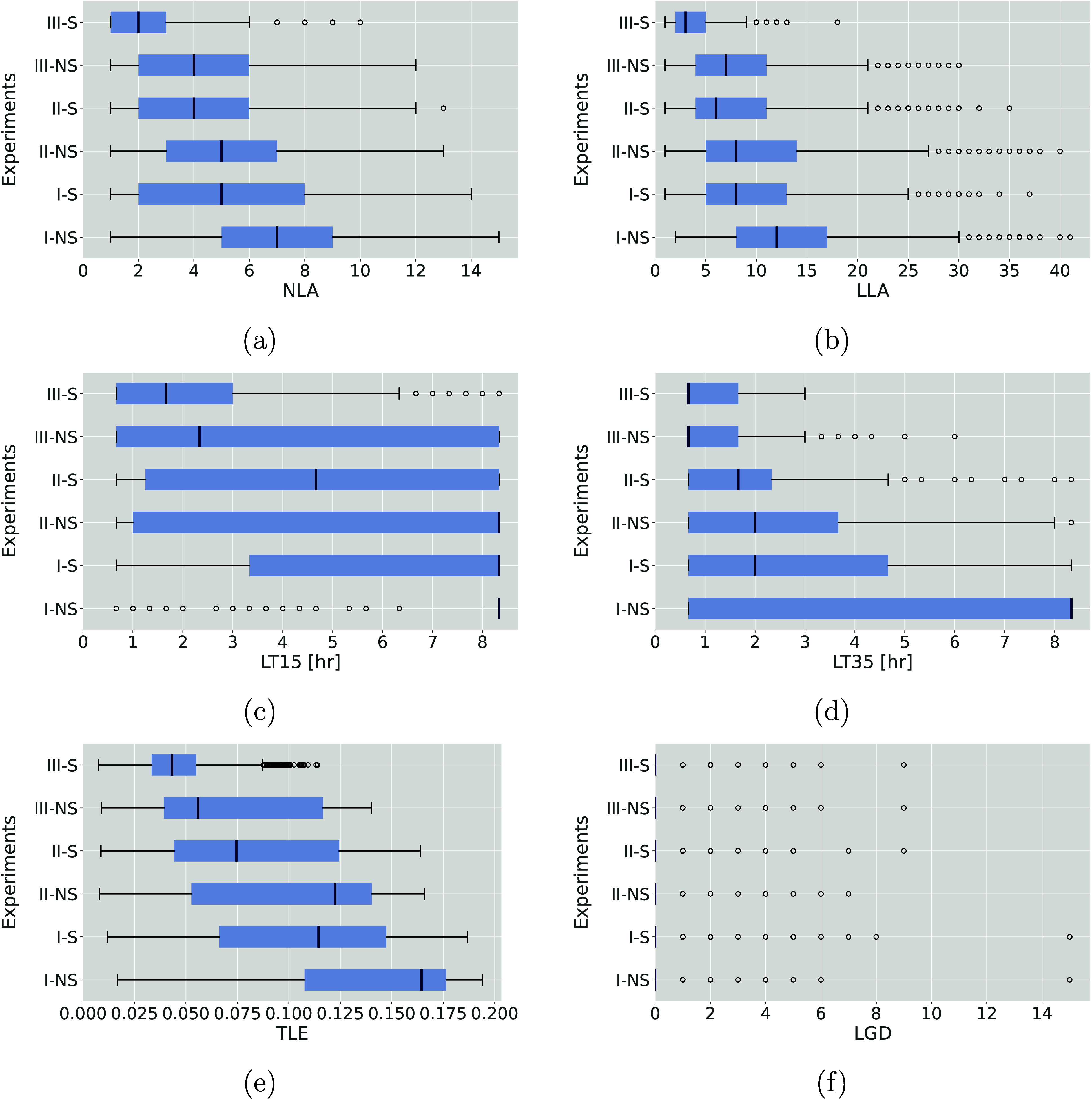
Distributions of (a) NLA, (b) LLA, (c) LT with *L*_*max*_ = 15, (d) LT with *L*_*max*_ = 35, (e) TLE, and (f) LGD with I, II, and III sensors and with (-S) and without (-NS) a sampler.

Next, we present a thorough analysis of the SL algorithm, focusing on performance across exhaustive scenarios with and without a sampler. We organize scenarios with injection start times varying at hourly intervals from 0:00 to 10:00, and sensor counts ranging from one to three, exploring a comprehensive range of network conditions. We conduct these simulations under both configurations, with groups using a sampler marked as ’-S’ and those without as ’-NS’. The results are then categorized into Groups I, II, and III, corresponding to the number of sensors used. Figure 8 shows boxplots with the distribution of the five performance metrics and localization times for *L*_*max*_ = 35 and *L*_*max*_ = 15.

#### Number and Size of Localization Areas

For the number and size of localization areas of potential sources, while NLA increases with samplers, their size, LLA, decreases. This is illustrated in Figure 8(a) and (b), where scenarios with samplers (I-S) show a greater median NLA than those without, indicating more potential localization areas. However, the median LLA value drops from 12 nodes in simpler setups (I-NS) to 7 in more complex configurations (III-NS), and further to 3 nodes in the most equipped setup (III-S). This demonstrates that while samplers increase the number of potential localization areas, they also break them into smaller, more precise zones.

#### Localization Times

We measure LT for *L*_*max*_ ∈ 35, 15, representing the time taken to reduce the number of probable sources to 35 and 15, respectively, and show the LT distribution across all scenarios in Figures 8(c) and (d). For *L*_*max*_ = 35, adding a sensor or sampler significantly reduces LT. With one fixed sensor (I-N), the median LT decreases from over 8 to 2 h when a sampler is added (I-S) and to under one hour with three sensors and one sampler (III-S) (Figure 8(d)). Although samplers do not reduce the median LT beyond the improvement achieved with one sensor, they effectively decrease the extreme values. Figure 8(c) indicates that achieving *L*_*max*_ = 15 is more challenging, necessitating additional fixed sensors. In cases of sparse spatial sensor coverage, samplers show greater benefits, reducing both the median and the spread of LT.

#### Localization Gap Distance

While sensors and samplers are generally effective in identifying the true source across most scenarios, there are cases where the method fails. Figure 8(f) shows the LGD distribution across different scenario groups, revealing outliers where LGD exceeds zero, even with samplers. These discrepancies are anticipated and primarily stem from model inaccuracies, which affect performance under heuristics H2 and H3. Even with an error tolerance of ε_*t*_ = 1 h, mismatches between simulated node states and actual concentration values can lead to the erroneous exclusion of the true source. This emphasizes the need to consider the accuracy in offline scenarios when calibrating the heuristics within the SL methodology. It also highlights the trade-off between precision and accuracy when using samplers, i.e., increasing the error margin enhances accuracy but may compromise precision.

#### Trade-offs between Sensors and Samplers

Analyzing the boxplots in Figure 8 reveals key performance differences and potential optimal combinations of sensors and samplers. Comparisons, such as I-S (one fixed sensor plus a sampler) versus II-NS (two fixed sensors without a sampler), suggest that adding a sampler may be more beneficial than simply adding another fixed sensor, as indicated by improved TLE metric (see Figure 8(d)). Similar trends are observed across the NLA, LLA, and LGD metrics, showing consistent performance improvements as the number of sensors and samplers increases, although results are mixed for other metrics. Overall, results indicate that samplers are particularly effective when integrated with a network of sensors, facilitating quicker and more accurate source localization. However, further research is needed to validate and generalize these results across various scenarios and water distribution systems.

### Sensitivity Analysis

Building on the results outlined above and considering various sources of uncertainty, we test the robustness of the proposed approach by evaluating its sensitivity to both initial sensor placement and the margin of uncertainty in time.

#### Sensitivity to Initial Sensor Placement

We first assess the sensitivity of the proposed approach to initial sensor placement, acknowledging multiple sources of uncertainty. These uncertainties include the reliance on a representative set of contamination scenarios that may not encompass all potential events, variations in sensor placement algorithms and objectives that lead to differing sensor locations, and practical constraints that may restrict sensor placement.^32,33^ To address this, we conduct a sensitivity analysis by testing eight different sensor placement configurations, evaluating all performance metrics across all exhaustive scenarios considering three sensors and a sampler (III-S). Configuration A represents the original sensor setup, while configurations B-H represent alternatives. These alternative setups are created by dividing the network into three zones and positioning one sensor at the centroid of each region. Results of this analysis are presented in Table S1 and Figure S13 in the Supporting Information.

Results indicate that NLA, LLA, and LGD metrics remain robust to variations in initial sensor configurations, while LT and TLE metrics are more sensitive. This implies that while adding samplers effectively identifies localization areas, the speed and precision of localization are significantly enhanced with optimal initial sensor placement.

#### Sensitivity to Uncertainty in Time of Arrival

Heuristics H2 and H3 involve comparing the sequence of observed arrival times while allowing for a margin of uncertainty, ε_*t*_. Scenarios where the expected arrival times fall outside this margin are discarded. The tolerance ε_*t*_ captures both model and measurement uncertainty, balancing between accuracy and precision, specifically, accurately identifying the localization zone of the true source versus minimizing the size of that zone. If ε_*t*_ is too large, H2 and H3 will fail to filter many scenarios, reducing precision. Conversely, if ε_*t*_ is too small, many potential sources could be erroneously excluded, including the true source, compromising accuracy.

We conduct a sensitivity analysis by varying ε_*t*_ from 0.5 to 3 h, evaluating all performance metrics across exhaustive scenarios with three sensors and a sampler (III-S). Results are shown in Figure S14 in the Supporting Information. Results reveal a tipping point at ε_*t*_ = 1 h. Beyond this value, metrics such as LLA, LT, and TLE deteriorate, indicating poorer performance in localization speed and effort. Conversely, when ε_*t*_ is lower than 1 h, NLA and LGD metrics worsen, suggesting that more events are incorrectly localized. This balance highlights the critical role of selecting an optimal ε_*t*_ to ensure both accuracy and precision in source localization.

## Conclusions

This work presents an SL methodology consisting of an online stage where sensor and sampler measurements are compared with offline simulation data using heuristics, resulting in a probability distribution of the most likely contamination sources. Our analysis demonstrates that incorporating samplers is crucial for enhancing precision, improving accuracy, and reducing localization times. Despite the potential benefits, the implementation of online water quality sensors in WDSs remains limited and is still evolving. Key barriers include concerns over sensor reliability, substantial upfront and maintenance costs, and the need for trained personnel to operate, maintain, and analyze sensor data. Since water utilities already perform periodic water quality grab sampling within WDSs, our findings show that these existing practices can be leveraged to significantly improve localization performance.

Although the SL method is effective for detecting and localizing the contamination sources, several limitations need to be addressed in future research. First, the reliability of the method using boolean and fuzzy sensor measurements remains uncertain.^31^ Further tests are needed to evaluate the impact of reduced sensor resolution, which could increase scenario ambiguity. Second, while offline simulations help reduce the online computational burden, the method’s scalability is limited when applied to networks with tens or hundreds of thousands of elements. For large-scale systems, localizing the source requires reducing the number of simulated scenarios to manage computational demands, which increases the risk of missing the true source. To ensure scalability, future work could explore using importance sampling^43^ to prioritize high-likelihood scenarios based on ongoing conditions, significantly reducing computational times by simulating only the most relevant cases. Third, additional sensitivity analyses are necessary to evaluate the impact of various settings, such as sampling delays and other sources of uncertainty. Additionally, future studies could investigate the effectiveness of using sampler data alone with heuristics H3 and H4 to determine the number of samplers needed to compensate for the absence of sensor data. While sensors and samplers complement each other, sensors are crucial for initial detection and fixed-location monitoring, and samplers refine localization by collecting data from diverse network locations, the optimal cost-benefit balance remains unclear. Although low-cost continuous-quality sensors are becoming more prevalent,^44^ it is still uncertain whether investments should prioritize expanding the sensor network or increasing the number of samplers. This work demonstrates that, in some scenarios, a sampler can provide performance improvements comparable to multiple sensors, suggesting that samplers may be a more cost-effective option when resources are limited.

## Supporting Information Available

Additional details and results for contaminant propagation, probability, and source localization update for example events; sensitivity analysis to the static sensor configuration; and sensitivity analysis to the time window parameter (PDF)

## References

[ref1] IAFP. Procedures to Investigate Waterborne Illness; Springer, 2016; pp 1–150.

[ref2] Danneels J. J.; Finley R. E. Assessing the vulnerabilities of US drinking water systems. Journal of contemporary water research and education 2004, 129, 8 10.1111/j.1936-704X.2004.mp129001003.x.

[ref3] Murray R.; Janke R.; Uber J. Critical transitions in water and environmental resources management. Environmental and Water Resources 2004, 2004, 1–8.

[ref4] Xin K.-l.; Tao T.; Li S.; Yan H. Contamination accidents in China’s drinking water distribution networks: status and countermeasures. Water Policy 2017, 19, 13–27. 10.2166/wp.2016.157.

[ref5] Pauli B. J. The Flint water crisis. Wiley Interdisciplinary Reviews: Water 2020, 7, e1420 10.1002/wat2.1420.

[ref6] Jacqueline C.; del Valle Arrojo M.; Bellver Moreira P.; Rodríguez Feijóo M. A.; Cabrerizo M.; Fernandez-Garcia M. D. Norovirus GII. 3 [P12] outbreak associated with the drinking water supply in a rural area in Galicia, Spain, 2021. Microbiology Spectrum 2022, 10, e01048-22 10.1128/spectrum.01048-22. 35867474 PMC9431064

[ref7] Perelman L.; Arad J.; Housh M.; Ostfeld A. Event detection in water distribution systems from multivariate water quality time series. Environ. Sci. Technol. 2012, 46, 8212–8219. 10.1021/es3014024. 22708647

[ref8] Mu T.; Huang M.; Tan H.; Chen G.; Zhang R. Pressure and water quality integrated sensor placement considering leakage and contamination intrusion within water distribution systems. ACS ES&T Water 2021, 1, 2348–2358. 10.1021/acsestwater.1c00209.

[ref9] Tiemann M. Safe Drinking Water Act (SDWA): A Summary of the Act and its Major Requirements; Congressional Research Service: Washington, DC, 2017.

[ref10] Laird C. D.; Biegler L. T.; van Bloemen Waanders B. G.; Bartlett R. A. Contamination source determination for water networks. Journal of Water Resources Planning and Management 2005, 131, 125–134. 10.1061/(ASCE)0733-9496(2005)131:2(125).

[ref11] Liu L.; Zechman E. M.; Mahinthakumar G.; Ranji Ranjithan S. Identifying contaminant sources for water distribution systems using a hybrid method. Civil Engineering and Environmental Systems 2012, 29, 123–136. 10.1080/10286608.2012.663360.

[ref12] Xuesong Y.; Jie S.; Chengyu H. Research on contaminant sources identification of uncertainty water demand using genetic algorithm. Cluster Computing 2017, 20, 1007–1016. 10.1007/s10586-017-0787-6.

[ref13] Butera I.; Gómez-Hernández J. J.; Nicotra S. Contaminant-source detection in a water distribution system using the ensemble Kalman filter. Journal of Water Resources Planning and Management 2021, 147, 04021029 10.1061/(ASCE)WR.1943-5452.0001383.

[ref14] Guan J.; Aral M. M.; Maslia M. L.; Grayman W. M. Identification of contaminant sources in water distribution systems using simulation–optimization method: case study. Journal of Water Resources Planning and Management 2006, 132, 252–262. 10.1061/(ASCE)0733-9496(2006)132:4(252).

[ref15] Eliades D. G.; Vrachimis S. G.; Moghaddam A.; Tzortzis I.; Polycarpou M. M. Contamination event diagnosis in drinking water networks: A review. Annual Reviews in Control 2023, 55, 420 10.1016/j.arcontrol.2023.03.011.

[ref16] Shang F.; Uber J. G.; Polycarpou M. M. Particle backtracking algorithm for water distribution system analysis. J. Environ. Eng. 2002, 128, 441–450. 10.1061/(ASCE)0733-9372(2002)128:5(441).

[ref17] Cai J.; Ye Z.-S. Contamination source identification: A Bayesian framework integrating physical and statistical models. IEEE Transactions on Industrial Informatics 2021, 17, 8189–8197. 10.1109/TII.2021.3062146.

[ref18] Ortega E.; Braunstein A.; Lage-Castellanos A. Contamination source detection in water distribution networks using belief propagation. Stochastic Environmental Research and Risk Assessment 2020, 34, 493–511. 10.1007/s00477-020-01788-y.

[ref19] Wang C.; Zhou S. Contamination source identification based on sequential Bayesian approach for water distribution network with stochastic demands. IISE transactions 2017, 49, 899–910. 10.1080/24725854.2017.1315782.

[ref20] Alnajim K.; Abokifa A. A. Bayesian Optimization for Contamination Source Identification in Water Distribution Networks. Water 2024, 16, 168 10.3390/w16010168.

[ref21] Shen Z.; Cao S.; Wang W.-X.; Di Z.; Stanley H. E. Locating the source of diffusion in complex networks by time-reversal backward spreading. Phys. Rev. E 2016, 93, 032301 10.1103/PhysRevE.93.032301. 27078360

[ref22] Mann A. V.; McKenna S. A.; Hart W. E.; Laird C. D. Real-time inversion in large-scale water networks using discrete measurements. Computers & chemical engineering 2012, 37, 143–151. 10.1016/j.compchemeng.2011.08.001.

[ref23] Perelman L.; Ostfeld A. Operation of remote mobile sensors for security of drinking water distribution systems. Water research 2013, 47, 4217–4226. 10.1016/j.watres.2013.04.048. 23764572

[ref24] Sankary N.; Oliker N.; Ostfeld A.; Rasekh A.; Wu R.; Banks M. K.; Porterfield M.. Mobile sensors for water quality management in water distribution systems. World Environmental and Water Resources Congress 2015; 2015, 792–801.

[ref25] Kazeminasab S.; Banks M. K. SmartCrawler: A Size-Adaptable In-Pipe Wireless Robotic System with Two-Phase Motion Control Algorithm in Water Distribution Systems. Sensors 2022, 22, 9666 10.3390/s22249666. 36560035 PMC9784413

[ref26] Shahmirnoori A.; Saadatpour M.; Rasekh A. Using mobile and fixed sensors for optimal monitoring of water distribution network under dynamic water quality simulations. Sustainable Cities and Society 2022, 82, 103875 10.1016/j.scs.2022.103875.

[ref27] Haxton T.; Klise K. A.; Laky D.; Murray R.; Laird C. D.; Burkhardt J. B. Evaluating Manual Sampling Locations for Regulatory and Emergency Response. Journal of water resources planning and management 2021, 147, 04021081 10.1061/(ASCE)WR.1943-5452.0001473. PMC9680873 36419672

[ref28] Wang H.; Harrison K. W. Bayesian approach to contaminant source characterization in water distribution systems: adaptive sampling framework. Stochastic environmental research and risk assessment 2013, 27, 1921–1928. 10.1007/s00477-013-0727-9.

[ref29] Besner M.-C.; Broséus R.; Lavoie J.; Giovanni G. D.; Payment P.; Prévost M. Pressure monitoring and characterization of external sources of contamination at the site of the Payment drinking water epidemiological studies. Environ. Sci. Technol. 2010, 44, 269–277. 10.1021/es901988y. 20039751

[ref30] Rodriguez J. S.; Bynum M.; Laird C.; Hart D.; Klise K.; Burkhardt J.; Haxton T. Optimal sampling locations to reduce uncertainty in contamination extent in water distribution systems. Journal of infrastructure systems 2021, 27, 04021026 10.1061/(ASCE)IS.1943-555X.0000628. PMC9628260 36330233

[ref31] Seth A.; Klise K. A.; Siirola J. D.; Haxton T.; Laird C. D. Testing contamination source identification methods for water distribution networks. Journal of Water Resources Planning and Management 2016, 142, 04016001 10.1061/(ASCE)WR.1943-5452.0000619.

[ref32] Hart W. E.; Murray R. Review of Sensor Placement Strategies for Contamination Warning Systems in Drinking Water Distribution Systems. Journal of Water Resources Planning and Management 2010, 136, 611–619. 10.1061/(ASCE)WR.1943-5452.0000081.

[ref33] Ostfeld A.; Uber J. G.; Salomons E.; Berry J. W.; Hart W. E.; Phillips C. A.; Watson J.; Dorini G.; Jonkergouw P.; Kapelan Z.; et al. The battle of the water sensor networks (BWSN): A design challenge for engineers and algorithms. Journal of Water Resources Planning and Management 2008, 134, 556–568. 10.1061/(ASCE)0733-9496(2008)134:6(556).

[ref34] Rossman L. A. EPANET 2: users manual; US Environmental Protection Agency, 2000.

[ref35] Klise K. A.; Murray R.; Haxton T. An Overview of the Water Network Tool for Resilience (WNTR); Sandia National Lab., Albuquerque, NM (United States), 2018.

[ref36] Berry J.; Hart W. E.; Phillips C. A.; Uber J. G.; Watson J.-P. Sensor placement in municipal water networks with temporal integer programming models. Journal of water resources planning and management 2006, 132, 218–224. 10.1061/(ASCE)0733-9496(2006)132:4(218).

[ref37] Klise K. A.; Nicholson B. L.; Laird C. D. Sensor placement optimization using Chama; 2017.

[ref38] Cormen T. H.; Leiserson C. E.; Rivest R. L.; Stein C. Introduction to algorithms; MIT Press, 2022.

[ref39] Guibert R.; Bayle A.; Plouraboué F. Geolocalization of water-waves origin within water distribution networks using time reversal of first event detection. Water Res. 2023, 230, 119538 10.1016/j.watres.2022.119538. 36587523

[ref40] Box G. E. P.; Jenkins G. M.; Reinsel G. C.; Ljung G. M. Time Series Analysis: Forecasting and Control, 5th ed.; Wiley, 2015.

[ref41] Backhaus J. Pareto Principle. Analyse & Kritik 1980, 2, 146–171. 10.1515/auk-1980-0203.

[ref42] Boccelli D. L. 03 PA 2. US Systems, 2016; https://uknowledge.uky.edu/wdst_us/3, Originally developed by Vasconcelos . in 1997, classified as a distribution branch by Hwang & Lansey (2017) and looped by Hoagland . (2015). Updated in October 2021.

[ref43] Tokdar S. T.; Kass R. E. Importance sampling: a review. Wiley Interdisciplinary Reviews: Computational Statistics 2010, 2, 54–60. 10.1002/wics.56.

[ref44] Pule M.; Yahya A.; Chuma J. Wireless sensor networks: A survey on monitoring water quality. Journal of applied research and technology 2017, 15, 562–570. 10.1016/j.jart.2017.07.004.

