# Supplementary material for: Heuristic Space Reduction Method for Source Localization in Water Distribution Networks
Source: ACS ES T Water. 2025 Feb 25;5(3):1099–111. doi: 10.1021/acsestwater.4c00671 (PMC11915354; doi:10.1021/acsestwater.4c00671)
Supplement: Supplementary file 1 — ew4c00671_si_001.pdf [file ew4c00671_si_001.pdf]

Supporting Information – Heuristic Space Reduction Meghod for Source Localization in Water Distribution Networks

Gerardo Riano-Briceno,[†] Ahmed Abokifa,[‡] Ahmad Taha,[¶] and Lina Sela[*,§]

†*Department of Civil, Architectural, and Environmental Engineering, The University of Texas at Austin, USA*

‡*Department of Civil, Materials, and Environmental Engineering, University of Illinois Chicago*

¶*Department of Civil and Environmental Engineering; Department of Electrical and Computer Engineering, Vanderbilt University*

§*Department of Civil, Architectural, and Environmental Engineering, The University of Texas at Austin*

E-mail: linasela@utexas.edu

Contents of this file

- Figures S1-S14

- Table S1

Results: Scenario B

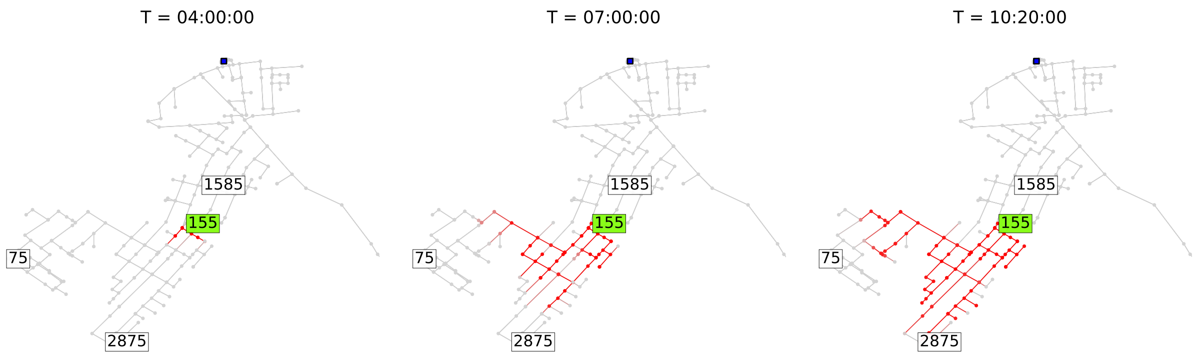

Figure S1: Propagation of contaminant in PA2 network for scenario B.

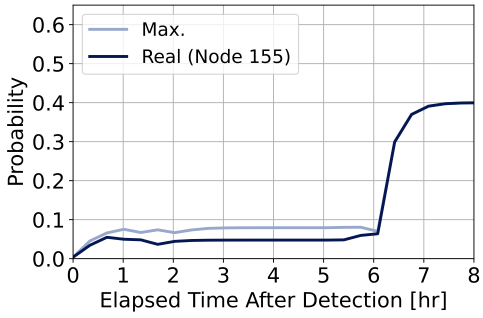

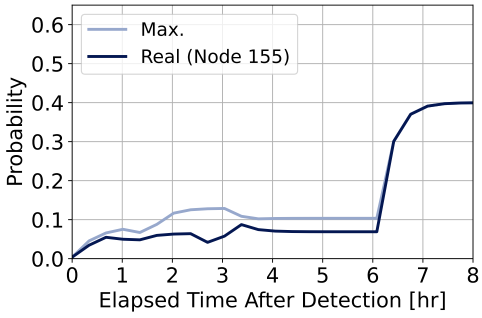

(a) (b)

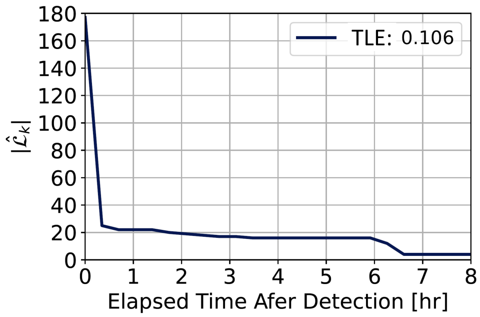

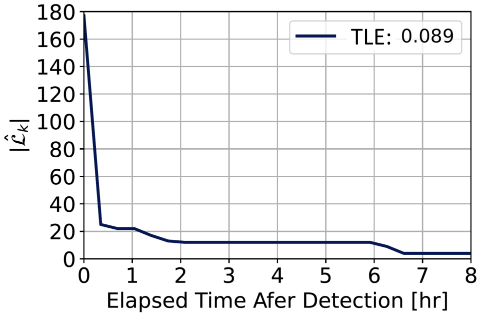

(c) (d)

Figure S2: Comparison of true source probability vs. maximum (a) with and (b) without a sampler in scenario B; (c) and (d) show the most probable nodes with and without a sampler.

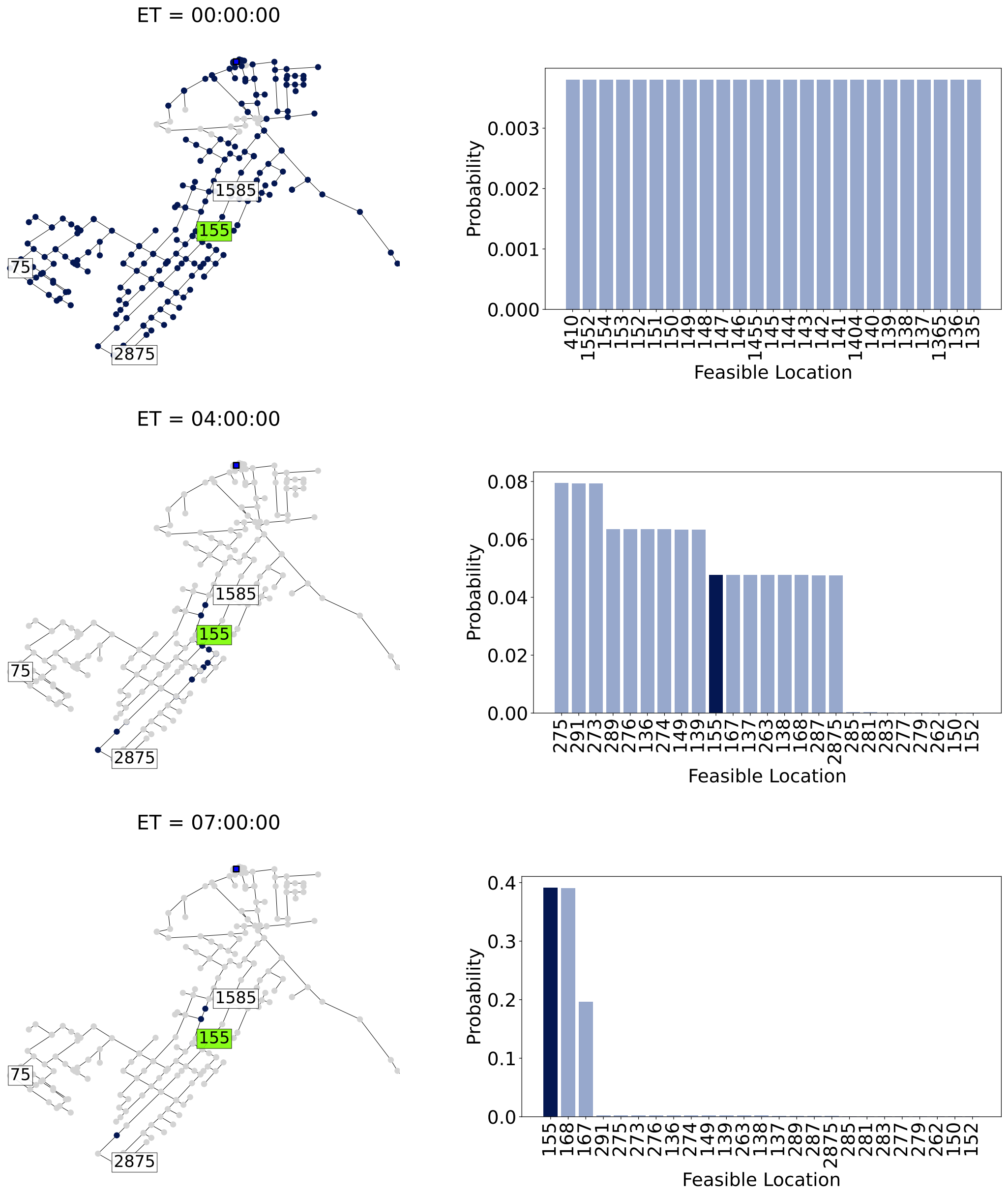

(a) (b)

Figure S3: Evolution of the estimation process with sensors for scenario B. (a) Shows feasible sources marked in dark blue, and (b) shows the probability of source, with the true source highlighted; only top 25 nodes are shown.

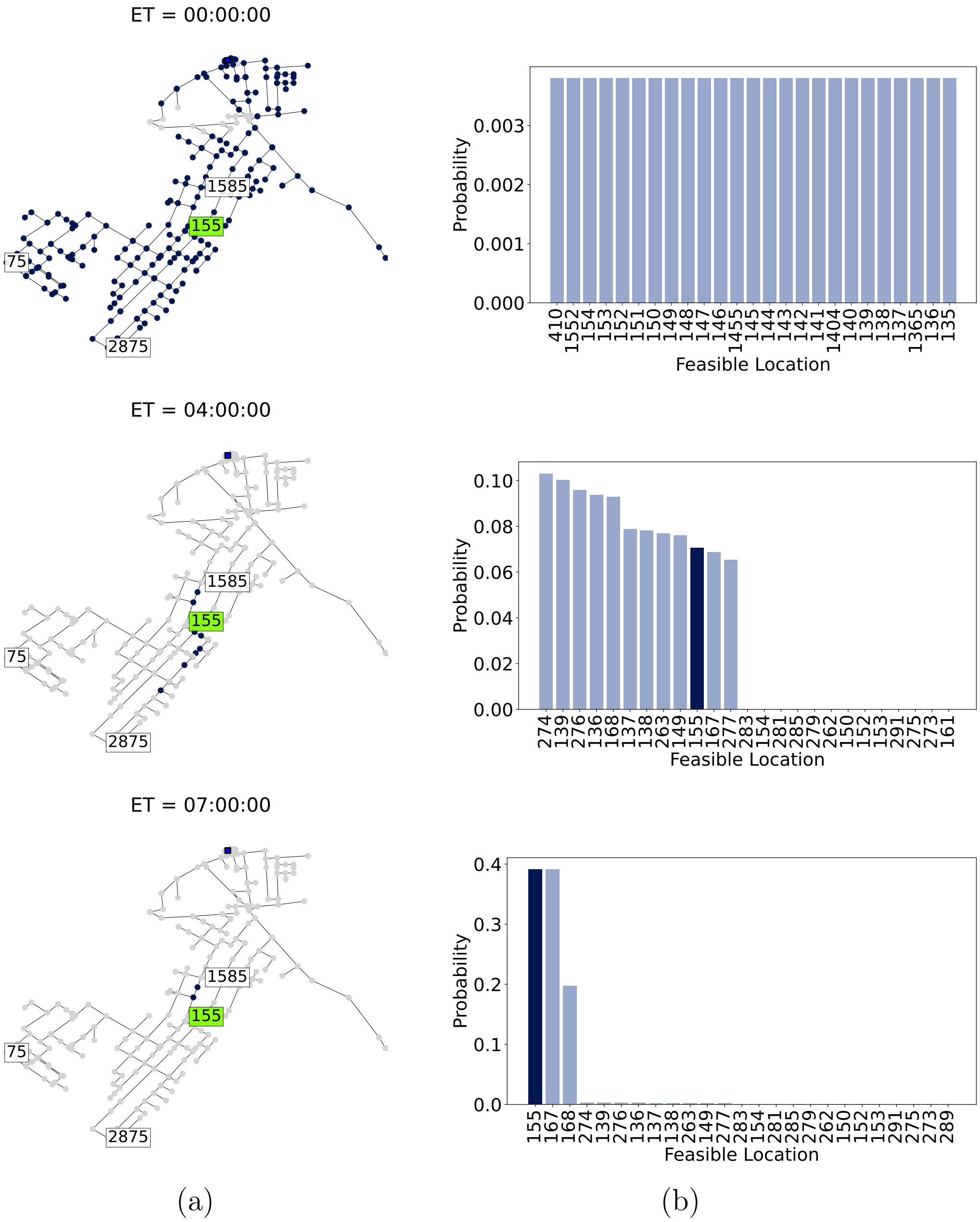

Figure S4: Evolution of the estimation process with sensors and samplers for scenario B. (a) Shows feasible sources marked in dark blue, and (b) shows the probability of source, with the true source highlighted; only top 25 nodes are shown.

Results: Scenario C

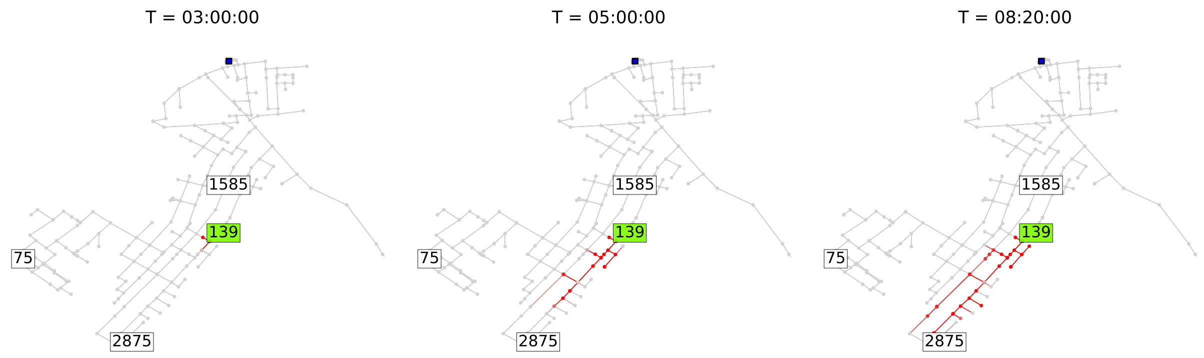

Figure S5: Propagation of contaminant in PA2 network for scenario C.

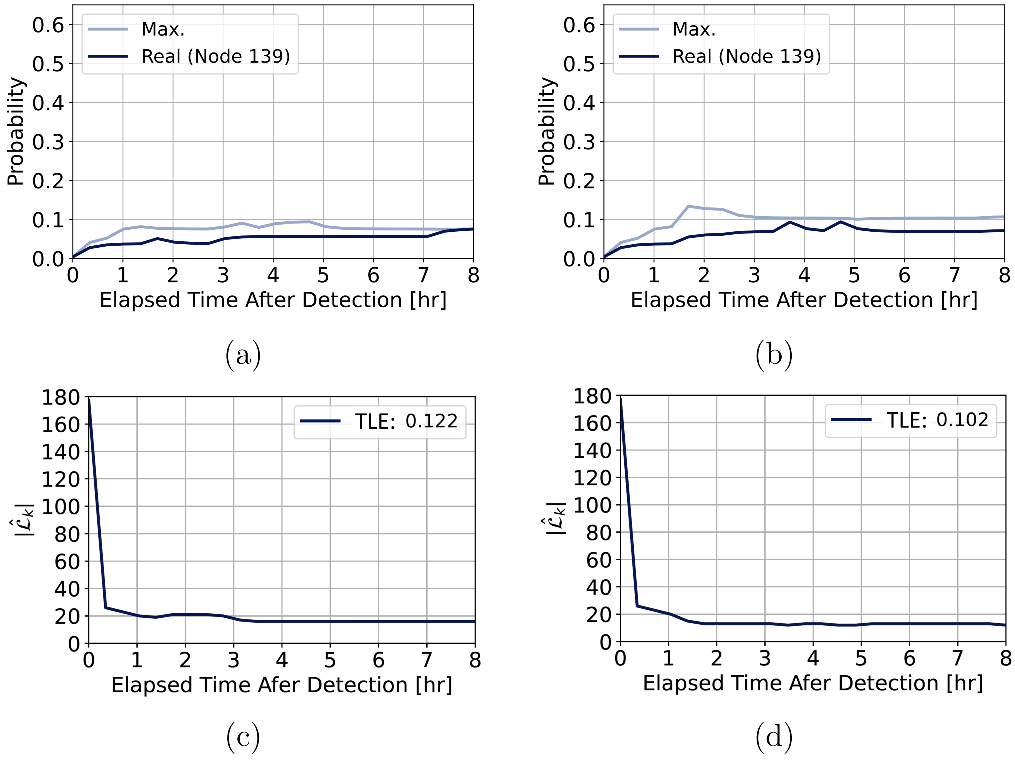

Figure S6: Comparison of true source probability vs. maximum (a) with and (b) without a sampler in scenario C; (c) and (d) show the most probable nodes without and without a sampler.

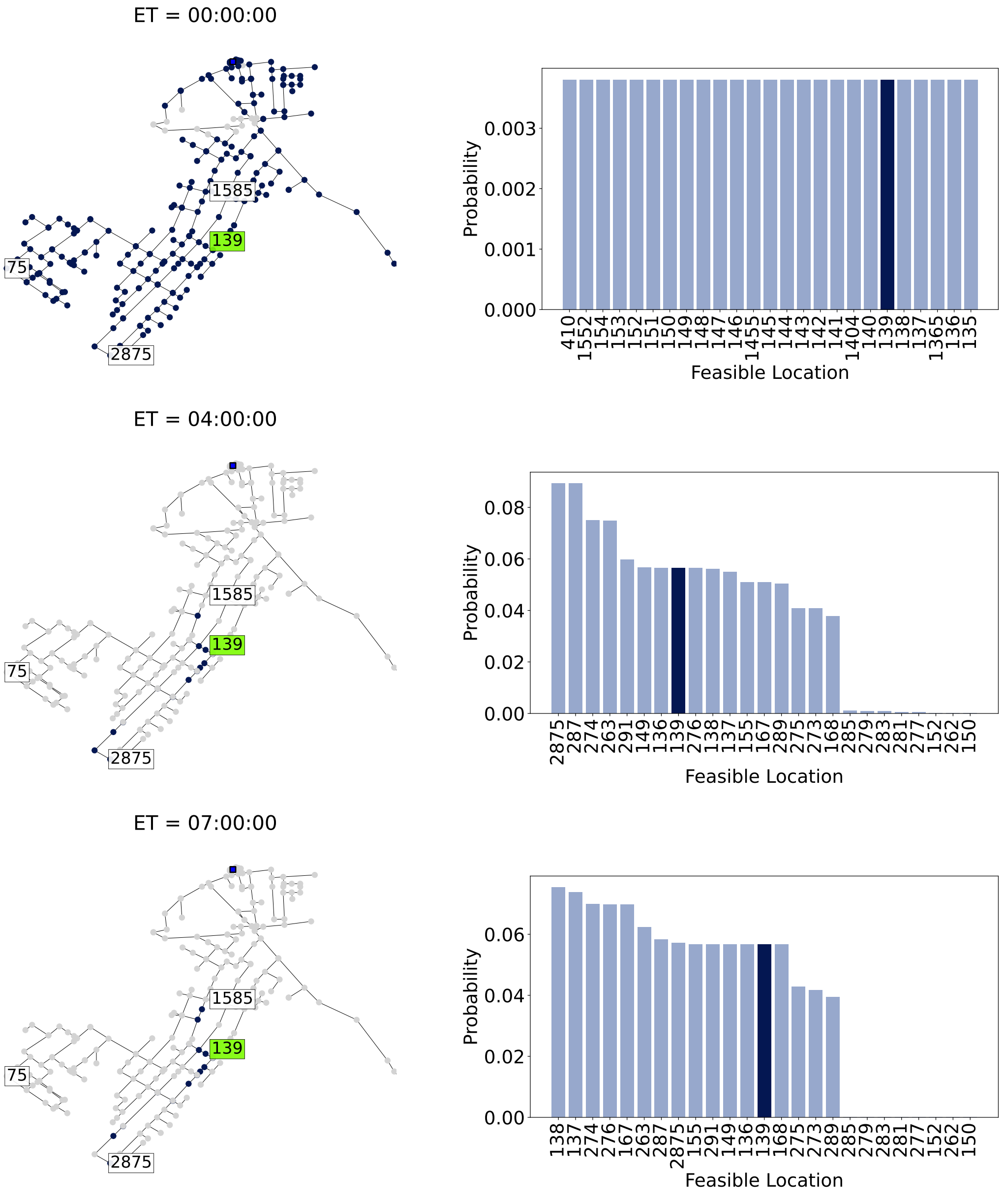

(a) (b)

Figure S7: Evolution of the estimation process with sensors for scenario C. (a) Shows feasible sources marked in dark blue, and (b) shows the probability of source, with the true source highlighted; only top 25 nodes are shown.

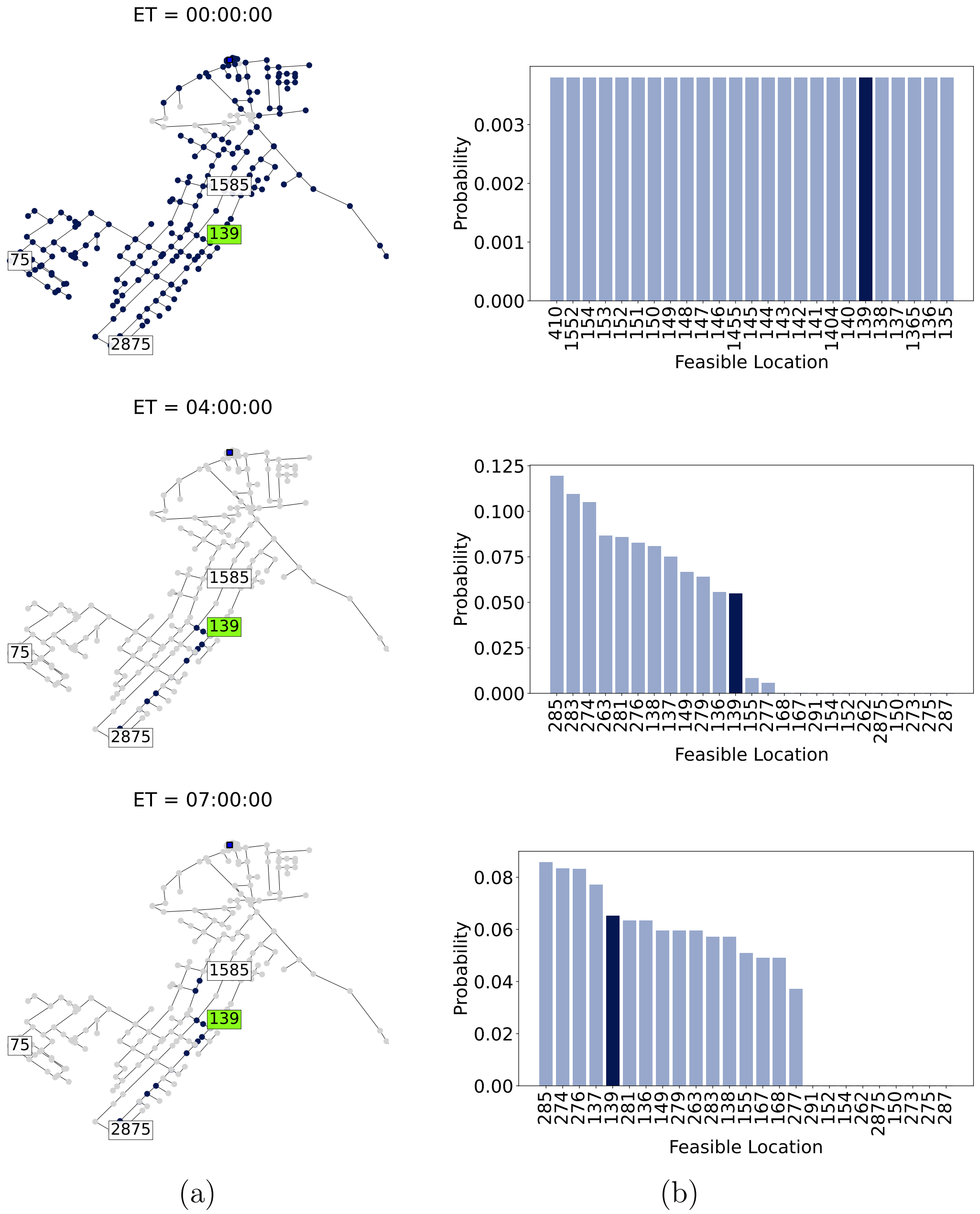

Figure S8: Evolution of the estimation process with sensors and sampler for scenario C. (a) Shows feasible sources marked in dark blue, and (b) shows the probability of source, with the true source highlighted; only top 25 nodes are shown.

Results: Scenario D

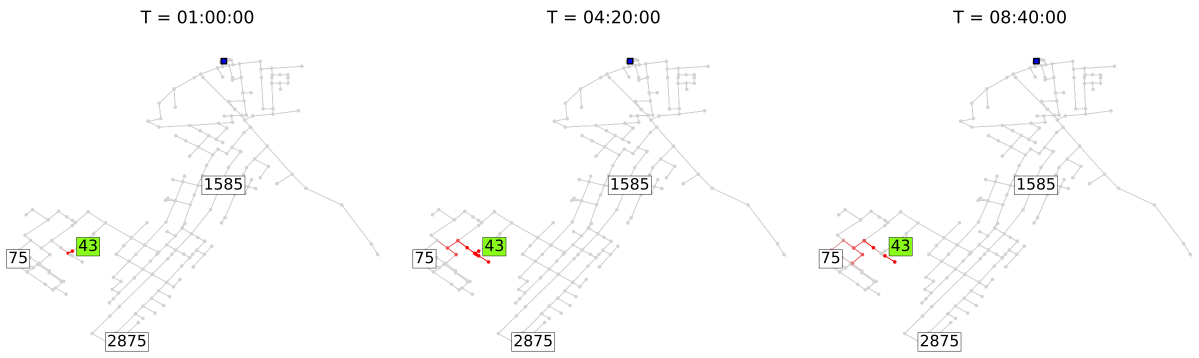

Figure S9: Propagation of contaminant in the PA2 network for select scenario D.

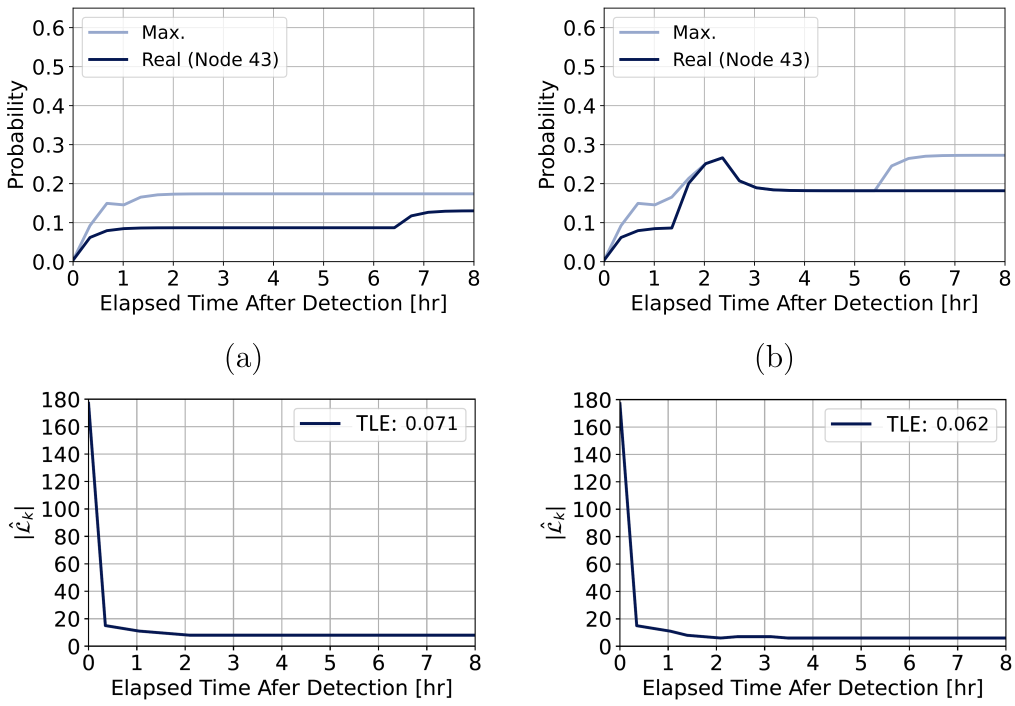

(a) (b)

(c) (d)

Figure S10: Comparison of true source probability vs. maximum (a) with and (b) without a sampler in select scenario D; (c) and (d) show the most probable nodes without and without a sampler; only top 25 nodes are shown.

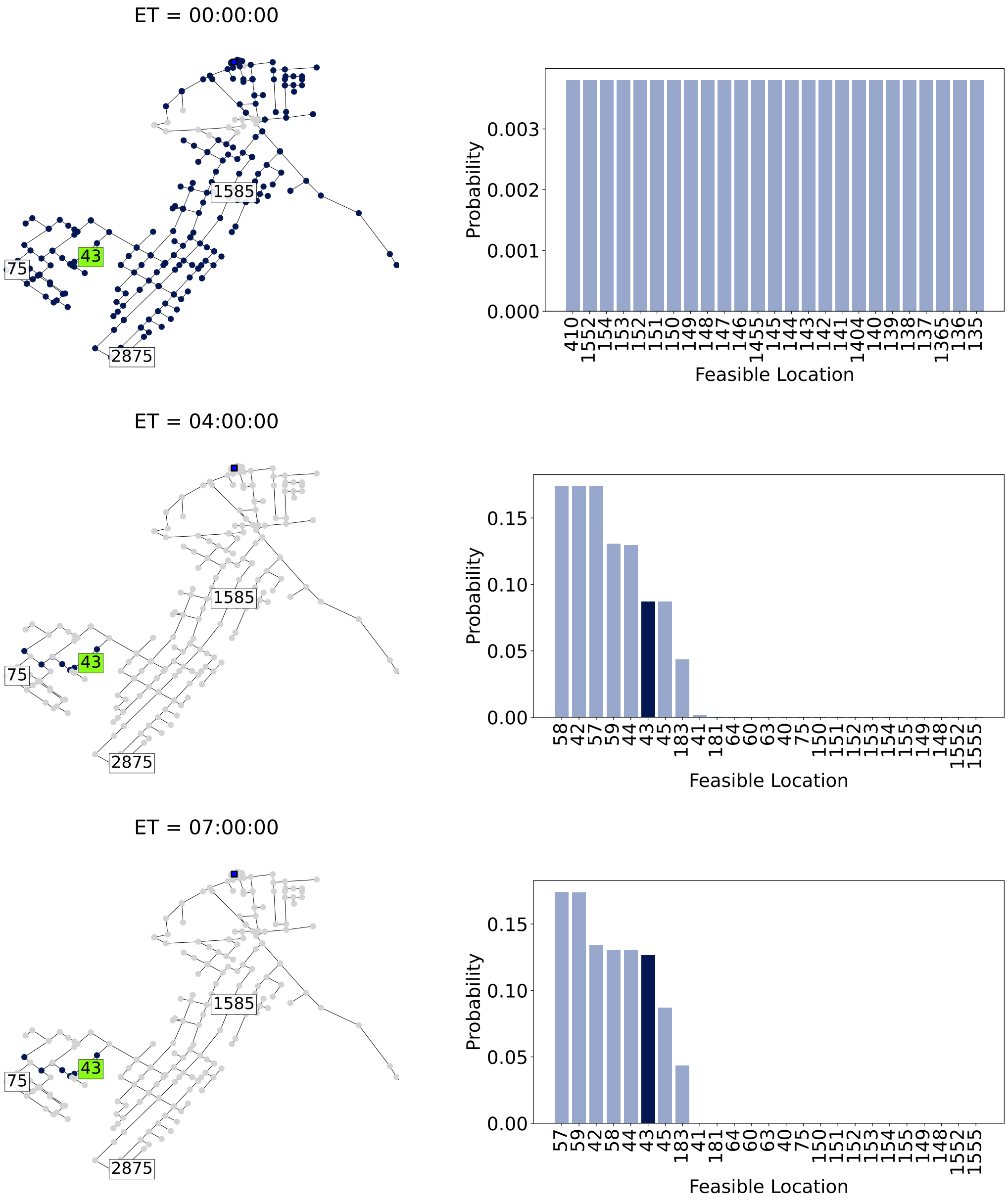

(a) (b)

Figure S11: Evolution of the estimation process with sensors for scenario D. (a) Shows feasible sources marked in dark blue, and (b) shows the probability of source, with the true source highlighted; only top 25 nodes are shown.

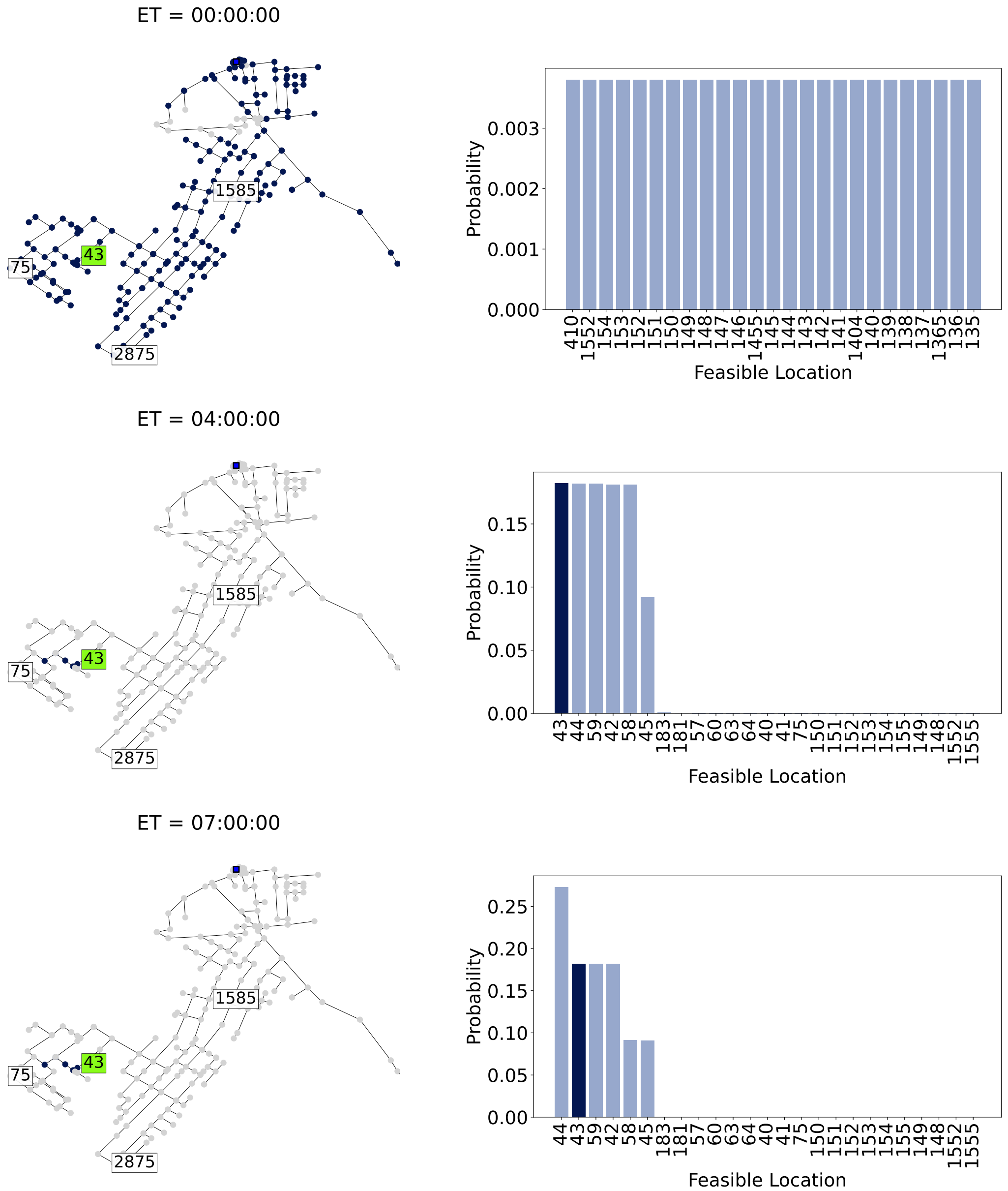

(a) (b)

Figure S12: Evolution of the estimation process with sensors for scenario D. (a) Shows feasible sources marked in dark blue, and (b) shows the probability of source, with the true source highlighted; only top 25 nodes are shown.

Sensitivity Analysis to Static Sensor Configuration

Table S1: Sensor Configurations Data

ID	Sensors	Num. Detectable Scenarios
S-A	'1585', '75', '2875'	1364
S-B	'1455', '291', '77'	1299
S-C	'163', '2512', '190'	957
S-D	'2265', '224', '1632'	946
S-E	'287', '124', '45'	1166
S-F	'2875', '184', '1845'	1166
S-G	'79', '215', '289'	1144
S-H	'805', '43', '244'	1210

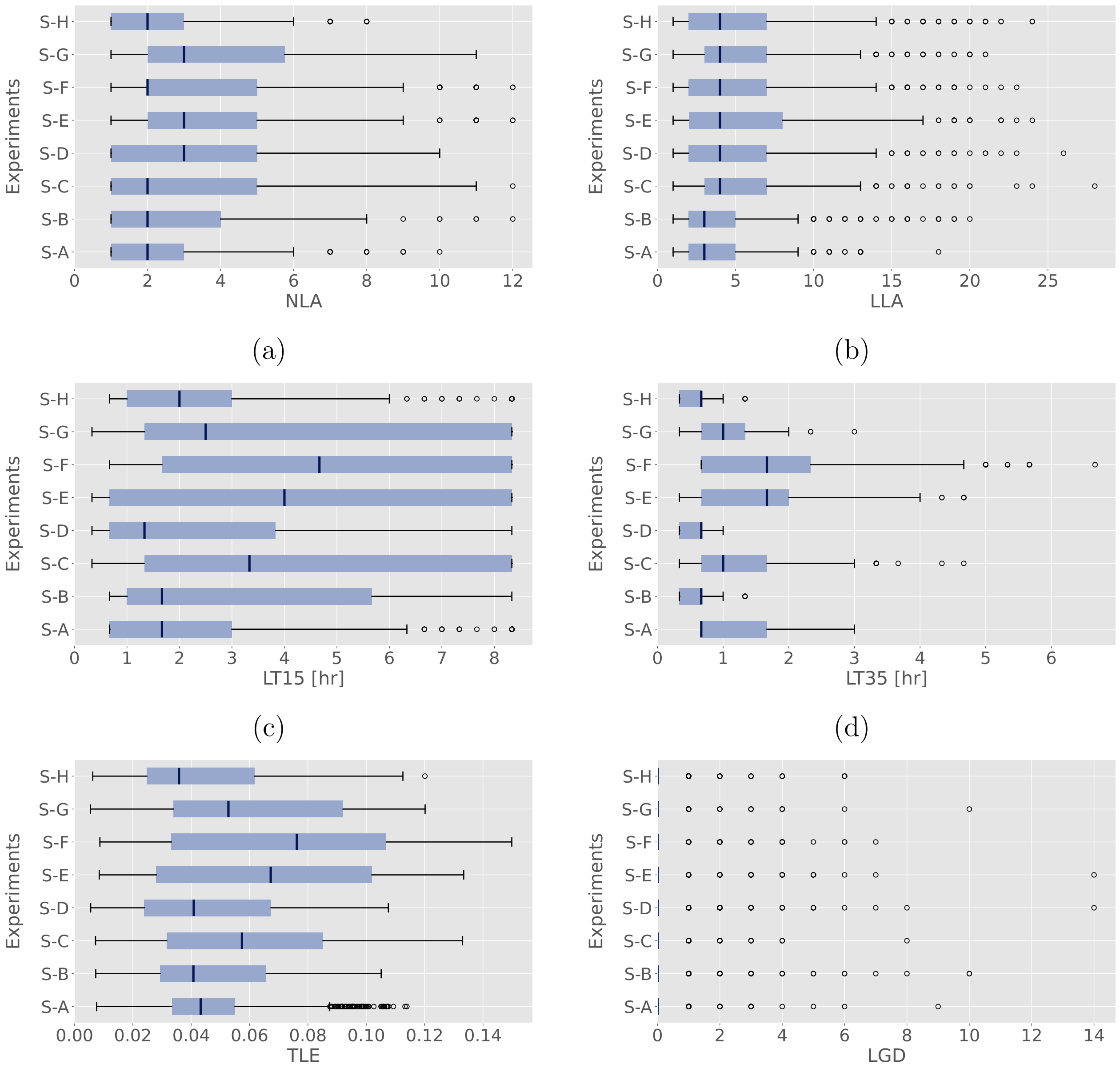

Figure S13: Distributions of (a) NLA, (b) LLA, (c) LT with $L_{max} = 15$, (d) LT with $L_{max} = 35$, (e) TLE, and (f) LGD for 6 exhaustive scenario runs varying sensor configurations, using 3 sensors and a sampler.

Sensitivity Analysis to Time Window Parameter

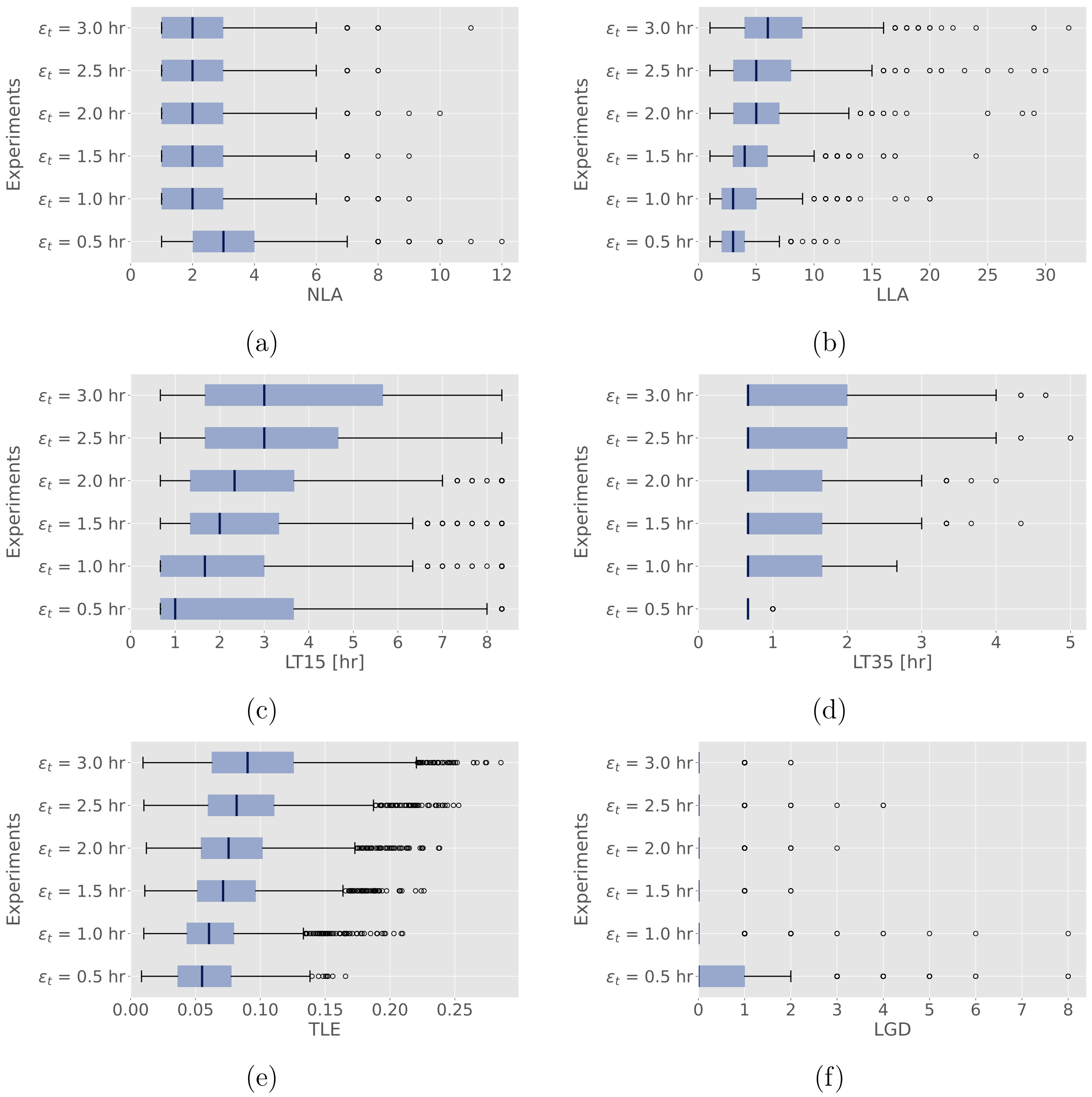

Figure S14: Distributions of (a) NLA, (b) LLA, (c) LT with $L_{max} = 15$, (d) LT with $L_{max} = 35$, (e) TLE, and (f) LGD for 6 exhaustive scenario runs varying ϵ_t between $[0.5, 1.0, 1.5, 2.0, 2.5, 3.0]$ hr using 3 sensors and a sampler.
